# Dose‐Dependent Reprogramming of Chromatin Accessibility by SOX4 Drives the Transcriptional Response to Iron Overload

**DOI:** 10.1002/advs.202521702

**Published:** 2026-05-07

**Authors:** Feifei Li, Yaoqiu Wu, Guangyu Yang, Jingyi Lai, Xiaoyue Sun, Liyan Wang, Xiaoli Li, Jing Zhang, Qingxue Zhang, Hui Chen, Haiyan Lin, Bingxiang Xu, Junfeng Zhang, Hailong Wang, Anming Meng, Chunwei Cao

**Affiliations:** ^1^ Division of Cell Developmental and Integrative Biology School of Medicine South China University of Technology Guangzhou China; ^2^ Shenzhen Maternity and Child Healthcare Hospital Women and Children's Medical Center Southern Medical University Shenzhen Guangdong China; ^3^ Guangdong Provincial Key Labo*r*atory of Malignant Tumor Epigenetics and Gene Regulation Guangdong‐Hong Kong Joint Laboratory for RNA Medicine Medical Research Center Sun Yat‐Sen Memorial Hospital Sun Yat‐Sen University Guangzhou China; ^4^ Guangzhou National Laboratory Guangzhou China; ^5^ Laboratory of Developmental Biology Department of Cell Biology and Genetics School of Basic Medical Sciences Chongqing Medical University Chongqing China; ^6^ Department of Obstetrics and Gynecology Sun Yat‐sen Memorial Hospital of Sun Yat‐Sen University Guangzhou China; ^7^ Key Laboratory of Hebei Province for Molecular Biophysics Institute of Biophysics School of Health Science & Biomedical Engineering Hebei University of Technology Tianjin China; ^8^ Laboratory of Molecular Developmental Biology State Key Laboratory of Membrane Biology Tsinghua‐Peking Center for Life Sciences School of Life Sciences Tsinghua University Beijing China

**Keywords:** 3D genome, cellular stress, chromatin accessibility, iron overload, SOX4

## Abstract

Iron overload induces cellular stress and is implicated in diverse pathological conditions. Nevertheless, the epigenetic mechanisms governing mammalian cellular responses to iron overload remain poorly characterized. Using multi‐omics profiling in human granulosa cells, we show that the transcriptional signature of iron‐stressed granulosa cells recapitulated that of granulosa cells from endometriosis patients. Mechanistically, iron excess triggered a time‐dependent, genome‐wide reduction in chromatin accessibility, which was associated with broad transcriptional suppression. Furthermore, fine‐scale chromatin looping underwent dynamic reorganization, concomitant with dysregulated expression of core iron metabolism genes. We identify SOX4 as a central, dosage‐sensitive regulator of this epigenetic reprogramming: its downregulation under iron stress drives chromatin compaction, while ectopic SOX4 expression largely restores accessibility. Moreover, SOX4 is directly regulated by TFEB through binding to the CLEAR motif in its promoter and, in turn, exerts its function by recruiting the SWI/SNF chromatin remodeling complex. Notably, SOX4 also mediated chromatin compaction under high‐androgen stimulation, suggesting its universal role as a stress‐responsive epigenetic regulator in granulosa cells. These results elucidate a TFEB‐SOX4‐SWI/SNF regulatory axis that orchestrates iron‐responsive chromatin plasticity, and more broadly, uncover SOX4 as a key mediator of chromatin adaptation to pathophysiological stressors, including iron overload and hyperandrogenism.

## Graphical abstract

This study demonstrates that iron overload triggers widespread chromatin compaction and transcriptional repression in human granulosa cells, recapitulating features of endometriosis. The epigenetic reprogramming is orchestrated by a TFEB‐SOX4‐SWI/SNF axis, with SOX4 acting as a central, dosage‐sensitive regulator.

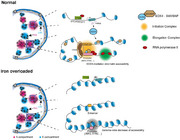

## 1 Introduction

Chronic stress exerts profound detrimental effects on human health by disrupting systemic homeostasis through multidimensional biological pathways [1, 2]. Under persistent environmental or pathological perturbations, cells orchestrate dynamic remodeling of intracellular signaling networks—an evolutionarily conserved survival strategy that coordinates stress adaptation across cellular, tissue, and organismal levels. This adaptive reprogramming engages synergistic interactions between epigenetic modifications, transcriptional rewiring, and translational regulation, collectively fine‐tuning stress‐responsive pathways [3, 4]. Notably, the chromatin architecture serves as a central regulatory hub, where stress‐induced alterations in chromatin topology, such as compartment switching, phase‐separated condensate formation, and loop domain reorganization, directly govern enhancer‐promoter connectivity [5, 6]. Such spatial genomic reconfigurations establish cell‐state‐specific transcriptional programs that underpin adaptive resilience, enabling organisms to dynamically recalibrate gene expression outputs in response to environmental challenges. Furthermore, cellular stress responses provide a paradigm for elucidating the bidirectional crosstalk between epigenetic memory systems and transcriptional machinery, revealing how nuclear chromatin organization integrates extracellular cues with genome‐wide regulatory circuits to maintain physiological equilibrium.

The causal interplay between chromatin architectural dynamics and transcriptional regulation remains a central enigma in epigenetics. Spatial chromatin interactions have been mechanistically implicated in orchestrating developmental gene activation cascades, as paradigmatically demonstrated by the spatiotemporal regulation of the β‐globin locus [7]. Intriguingly, depletion of key architectural proteins (e.g., CTCF/cohesin complex) substantially disrupts higher‐order chromatin folding, yet global transcriptional profiles exhibit remarkable resilience under such perturbations [8, 9]. This paradox highlights the existence of context‐dependent buffering mechanisms decoupling structural integrity from transcriptional homeostasis. Cellular stress responses emerge as a powerful paradigm to dissect this complexity, as they trigger rapid chromatin reorganization and stimulus‐specific transcriptional reprogramming. For instance, hypertonic stress triggers multi‐scale 3D genome destabilization, sequentially disrupting A/B compartments, topologically associating domain (TAD), and chromatin loops [10]. Hypoxic stress triggers chromatin accessibility changes mediated by HIF‐dependent nucleosome repositioning, preceding detectable transcriptomic shifts [11]. Thermal stress exhibits temporal specificity: acute heat shock preserves chromatin accessibility but redistributes transcriptional resources through phase‐separated stress granules [12], whereas chronic hyperthermia alters 3D genome conformation via cell cycle arrest‐induced chromatin compaction [13]. These observations delineate a stimulus‐encoded regulatory logic wherein chromatin topological remodeling and transcriptional reprogramming are dynamically negotiated to achieve context‐optimized cellular adaptation.

Iron serves as an essential cofactor in fundamental biological processes, including oxygen transport and mitochondrial oxidative phosphorylation, yet exhibits dose‐dependent cytotoxicity via Fenton reaction‐mediated oxidative stress [14, 15]. Cellular iron homeostasis is predominantly governed by the IRP‐IRE (Iron Regulatory Protein‐Iron Responsive Element) axis [16, 17]. Under iron‐deficient conditions, IRP1 and IRP2 bind IRE stem‐loop structures in target mRNAs: stabilizing transferrin receptor 1 (TFRC) transcripts to enhance iron uptake while blocking ferritin translation to limit iron sequestration. Conversely, iron‐replete conditions trigger IRP1's conversion to a cytosolic aconitase through 4Fe‐4S cluster assembly, abolishing its RNA‐binding capacity, while promoting FBXL5‐mediated ubiquitination and proteasomal degradation of IRP2. Iron overload represents a clinically significant etiological factor in diverse pathological states. Notably, in endometriosis‐associated infertility, ectopic endometrial lesions undergo cyclic hemorrhage within ovarian follicles, generating supra‐physiological iron concentrations in follicular fluid. This iron excess drives granulosa cell dysfunction and critically impairs oocyte developmental competence [18]. While transcriptomic studies reveal iron‐dependent gene expression alterations across cell types, the epigenetic dimension, particularly how chromatin architecture senses iron flux to orchestrate transcriptional adaptation, remains elusive. Furthermore, under iron overload conditions, the potential involvement of additional regulatory nodes, particularly chromatin‐based epigenetic modifiers, in modulating IRP activity and iron homeostasis remains to be systematically elucidated.

To address these questions, we established an iron‐overloaded granulosa cell model and performed comprehensive multi‐omics analyses, including transcriptional profiling, chromatin accessibility assessment, histone modification, and core architectural protein mapping, as well as 3D genome structure characterization, under high‐iron conditions. Our findings revealed that chromatin accessibility underwent widespread remodeling, which was closely associated with transcriptional changes under iron overload. Despite the stability of large‐scale compartments and topologically associating domains (TADs), fine‐scale chromatin loops were reprogrammed, including those involving key iron metabolism‐associated genes. Mechanistically, SOX4 acts as a dosage‐sensitive master regulator of iron‐induced epigenetic reprogramming. Under iron stress, binding of TFEB to the CLEAR motif in the SOX4 promoter is attenuated, resulting in decreased SOX4 expression. Consequently, SOX4‐dependent recruitment of the SWI/SNF chromatin remodeling complex is reduced, contributing to widespread chromatin condensation. Ectopic expression of SOX4 effectively rescues chromatin accessibility. Strikingly, high‐androgen stimulation phenocopies iron overload, inducing SOX4 loss and chromatin closure, thereby positioning SOX4 as a shared epigenetic effector of diverse pathophysiological stresses in granulosa cells. These results highlight stress‐induced changes in chromatin structure, uncover a novel epigenetic layer of iron homeostasis regulation that operates in parallel to the classical IRP‐IRE system, and underscore the central role of TFEB‐SOX4‐SWI/SNF axis in mediating chromatin responses to diverse pathological stimuli.

## 2 Results

### 2.1 Iron‐Overload Triggers Pervasive Transcriptional Reprogramming in Granulosa Cells

**FIGURE 1:**
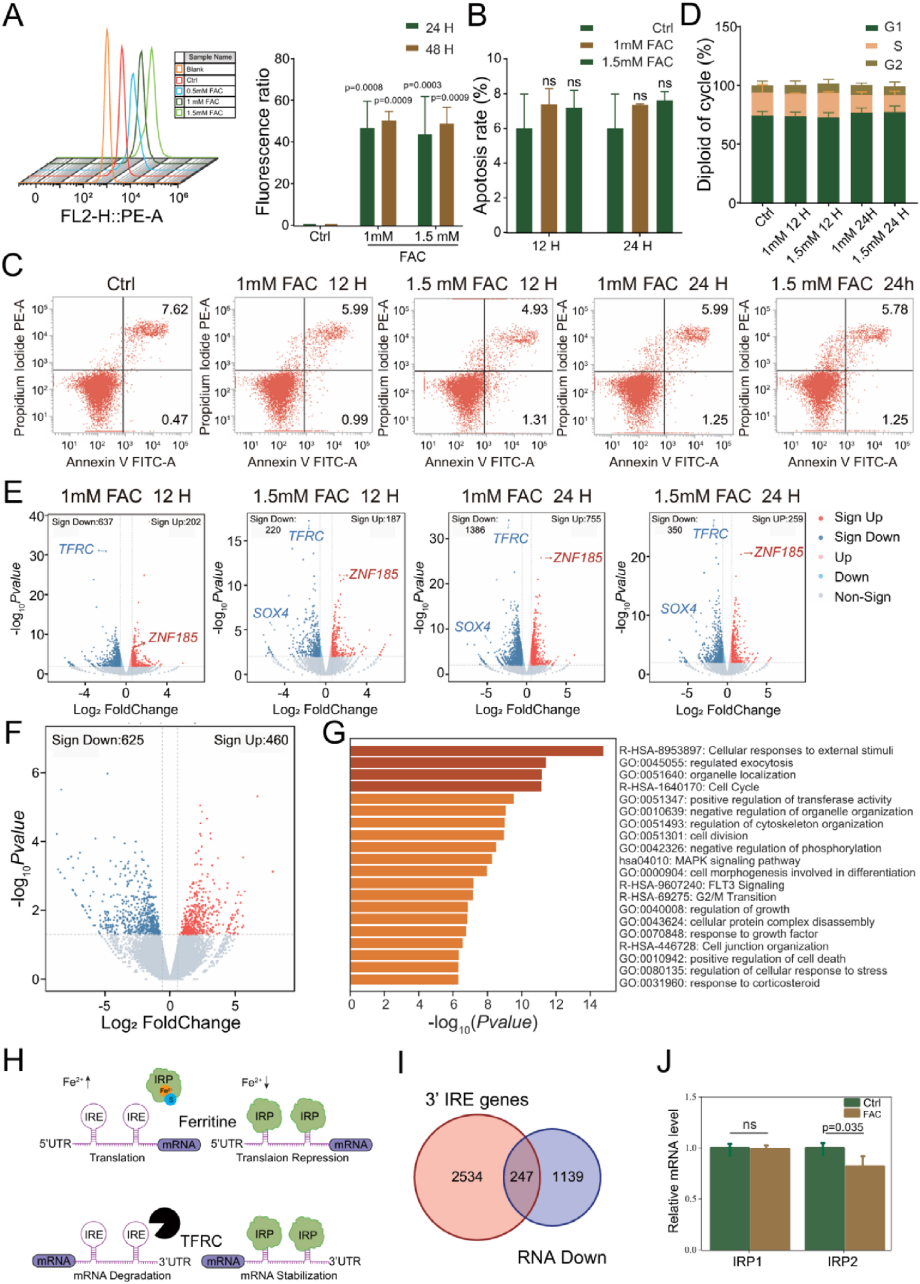
Iron‐overload significantly altered the gene expression profile in KGN cells. (A) Representative fluorescence‐density histograms (FeRhoNox‐1, FL2‐H: PE‐A) showing intracellular iron levels in control cells (Ctrl) and cells treated with 0.5mM, 1 mM or 1.5 mm FAC. Mean fluorescence intensity (MFI) quantified from n = 3 biological replicates (mean ± SD). Statistical significance was determined by an unpaired two‐tailed Student's *t*‐test. (B) Cell apoptosis analysis of KGN cell after FAC treatment with different concentrations and time durations. Each experiment was repeated three times, and the results are presented as means ± SD. Statistical significance was determined by an unpaired two‐tailed Student's *t*‐test. Statistical notation used throughout the figure: ns, not significant; ^*^ *p* < 0.05; ^**^ *p* < 0.01; ^***^ *p* < 0.001. (C) Flow cytometric analysis displays the proportion of apoptotic cells. (D) Cell cycle distribution of cells with different FAC treatment conditions (*p* > 0.05 for all comparisons). Each experiment was repeated three times and the results are presented as means ± SD. Statistical significance was determined by an unpaired two‐tailed Student's *t*‐test. (E) The differentially expressed genes between control and FAC treated cells were shown using a volcano plot. (F) Differentially expressed genes between granulosa cells in the affected and contralateral ovaries of an EM patients were shown using a volcano plot. (G) Pathway enrichment analysis of the upregulated genes after FAC treatment. (H) Schematic diagram showing the IRP‐IRE regulatory axis under iron stress. (I) Overlap between down‐regulated genes in RNA‐seq and genes containing the IRE element in their 3’ UTR. (J) qPCR validation of IRP1 and IRP2 expression after FAC treatment. Data are presented as mean ± SD from three independent biological replicates; *p* = 0.035 for *IRP2* vs. control (unpaired two‐tailed Student's *t*‐test).

To recapitulate the iron‐rich microenvironmental characteristic of granulosa cells in endometriosis (EM), we established an iron‐overload model using the KGN granulosa cell line. Guided by our previous findings, wherein the follicular fluid of EM patients exhibited an iron concentration of approximately 1.32 mm [19], we treated KGN cells with ferric ammonium citrate (FAC) at concentrations of 1.0 and 1.5 mm, aiming to closely replicate the pathological condition. Intracellular total iron levels revealed a significant increase in iron content following FAC exposure (Figure 1A). Moreover, treatment with iron for 12 h and 24 h does not compromise cell viability (Figure 1B,C) or alter cell cycle (Figure 1D; Figure S1A). Collectively, these results demonstrate that FAC‐treated KGN cells effectively model the iron‐overload stress experienced by granulosa cells in EM.

To delineate iron overload‐induced transcriptional reprogramming in granulosa cells, we conducted comprehensive RNA sequencing across four experimental conditions: KGN cells were treated with FAC at either 1.0 or 1.5 mm for either 12 or 24 h. FAC exposure significantly altered the transcriptional profile of KGN cells (Figure 1E). TFRC emerged as the most significantly downregulated gene across all treatment conditions (Figure 1E; Figure S1B), aligning with its canonical role in cellular iron uptake and suggesting activation of iron‐sensing feedback mechanisms. Transcriptional perturbations exhibited pronounced temporal amplification: the number of differentially expressed genes (DEGs) increased from 839 to 2141 (+155%) at 1.0 mm and from 407 to 609 (+49.6%) at 1.5 mm between 12 and 24 h. Notably, transcriptional repression dominated all experimental groups, with downregulated genes outnumbering upregulated counterparts by 1.18–3.15 fold, mirroring the gene suppression patterns detected in granulosa cells from EM patients (Figure 1F). Comparative transcriptomic profiling of matched affected and contralateral ovarian samples from EM patients identified 1085 DEGs, demonstrating analogous downregulation dominance (625 decreased vs. 460 increased), thereby validating the clinical relevance of our iron overload model. Additionally, upregulated genes demonstrated significant enrichment in “Cellular Responses to External Stimuli” and “Exocytosis” pathways (FDR < 0.05), mechanistically aligning with granulosa cells' adaptive reprogramming under iron overload (Figure 1G). Together, these findings demonstrate that iron overload reshapes the granulosa cell transcriptome primarily through a genome‐wide bias toward transcriptional repression. Given the maximal transcriptional perturbation observed in the 1.0 mm FAC 24 h group, this treatment paradigm was selected for subsequent mechanistic investigation. Furthermore, the canonical IRP/IRE regulatory axis is well‐established to mediate transcriptional repression through 3′UTR‐dependent mRNA destabilization under iron stress (Figure 1H). However, bioinformatic prediction revealed only 17.8% of FAC‐induced downregulated genes contained consensus IRE motifs within their 3′UTRs (Figure 1I). Interestingly, our investigation demonstrated that the pivotal iron regulatory factor IRP2, rather than IRP1, exhibited significantly reduced transcriptional activity under the iron overload conditions (Figure 1J). These results strongly indicate the existence of novel regulatory mechanisms operating beyond the canonical IRP/IRE paradigm.

### 2.2 The Nuclear Lamina Remained Intact during Iron‐Overload Stress and was Not Responsible for the Transcriptional Reprogramming

**FIGURE 2:**
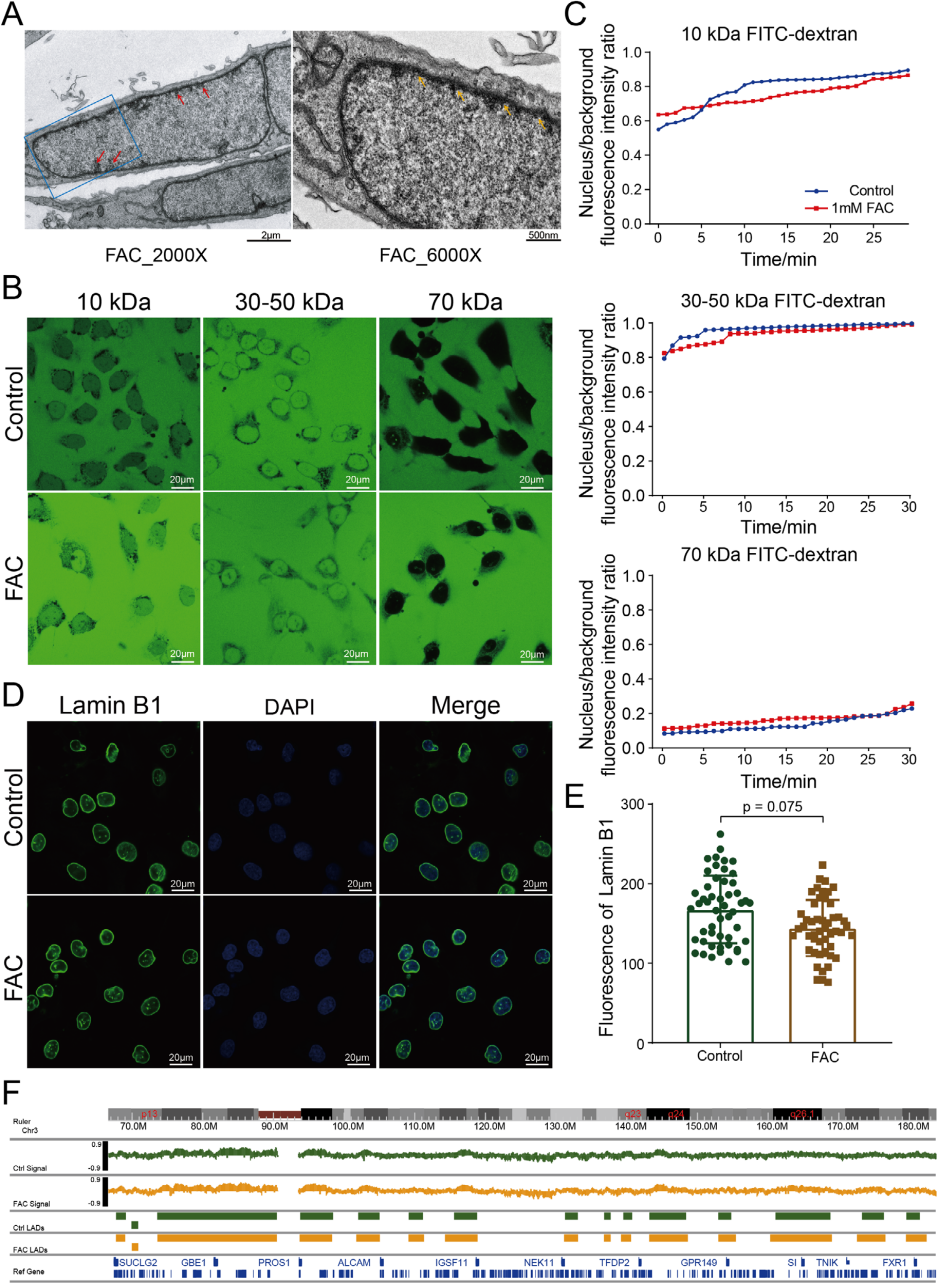
The nuclear lamina remained intact during iron‐overload stress. (A) Transmission Electron Microscopy (TEM) images of FAC‐treated KGN cells. The area outlined in blue indicates the region enlarged in the right panel. Condensed, dark‐stained chromatin aggregates are marked by red arrows, and the blurred nuclear envelope is indicated by yellow arrows. (B) Images showing the importation of FITC‐dextran conjugates with different molecular weights in both control and FAC‐treated cells. (C) Quantification of intracellular fluorescence intensity in (B). (D) Immunofluorescence detection of Lamin B1 and DAPI. (E) Quantitative comparison of Lamin B1 fluorescence intensity under the indicated conditions. Data are presented as mean ± SD from 50 cells per condition (two‐tailed unpaired *t*‐test, p = 0.075). (F) Lamin B1 ChIP‐seq signal and detected LADs in control and FAC‐treated cells, taking chr3: 70–180 Mb as an example.

To mechanistically dissect iron overload‐induced transcriptional reprogramming at chromatin resolution, we conducted ultrastructural analysis of FAC‐treated granulosa cells. Transmission electron microscopy revealed focal disruptions of the nuclear envelope in FAC‐treated cells, characterized by the unclarity of the double nuclear membrane (Figure 2A; Figure S2A). Given the established role of the nuclear lamina (NL) in maintaining heterochromatin organization through anchoring to the inner nuclear membrane interface, these structural perturbations at NL‐chromatin contact zones raised the possibility that iron excess might perturb higher‐order chromatin architecture, potentially contributing to the observed genome‐wide transcriptional repression.

However, comprehensive nuclear integrity assessments revealed preserved nuclear lamina architecture and function under iron‐overload stress. Nuclear permeability assays showed efficient import of small FITC‐dextran conjugates (10 kDa; 30–50 kDa) while maintaining strict exclusion of 70‐kDa macromolecules in both control and FAC‐treated cells (Figure 2B,C), confirming intact selective barrier function of the nuclear envelope. Immunofluorescence colocalization analysis of Lamin B1 and DAPI staining maintained precise localization at the nuclear periphery (Figure 2D,E), corroborated by unaltered Lamin B1 protein levels (Figure S2B,C). Critically, Lamin B1 ChIP‐seq further confirmed the conservation of genome‐wide lamina‐associated domain (LAD) architecture after FAC treatment, with minimal changes in both the number and length distribution of LADs (Figure 2F, Figure S2D,E). These lines of evidence definitively demonstrate nuclear lamina kept intact and is therefore unlikely to underlie the widespread transcriptional reprogramming observed in FAC‐treated granulosa cells.

### 2.3 Genome‐Wide Decrease of Chromatin Accessibility was Associated with Transcriptional Reprogramming Under Iron‐Overload

**FIGURE 3:**
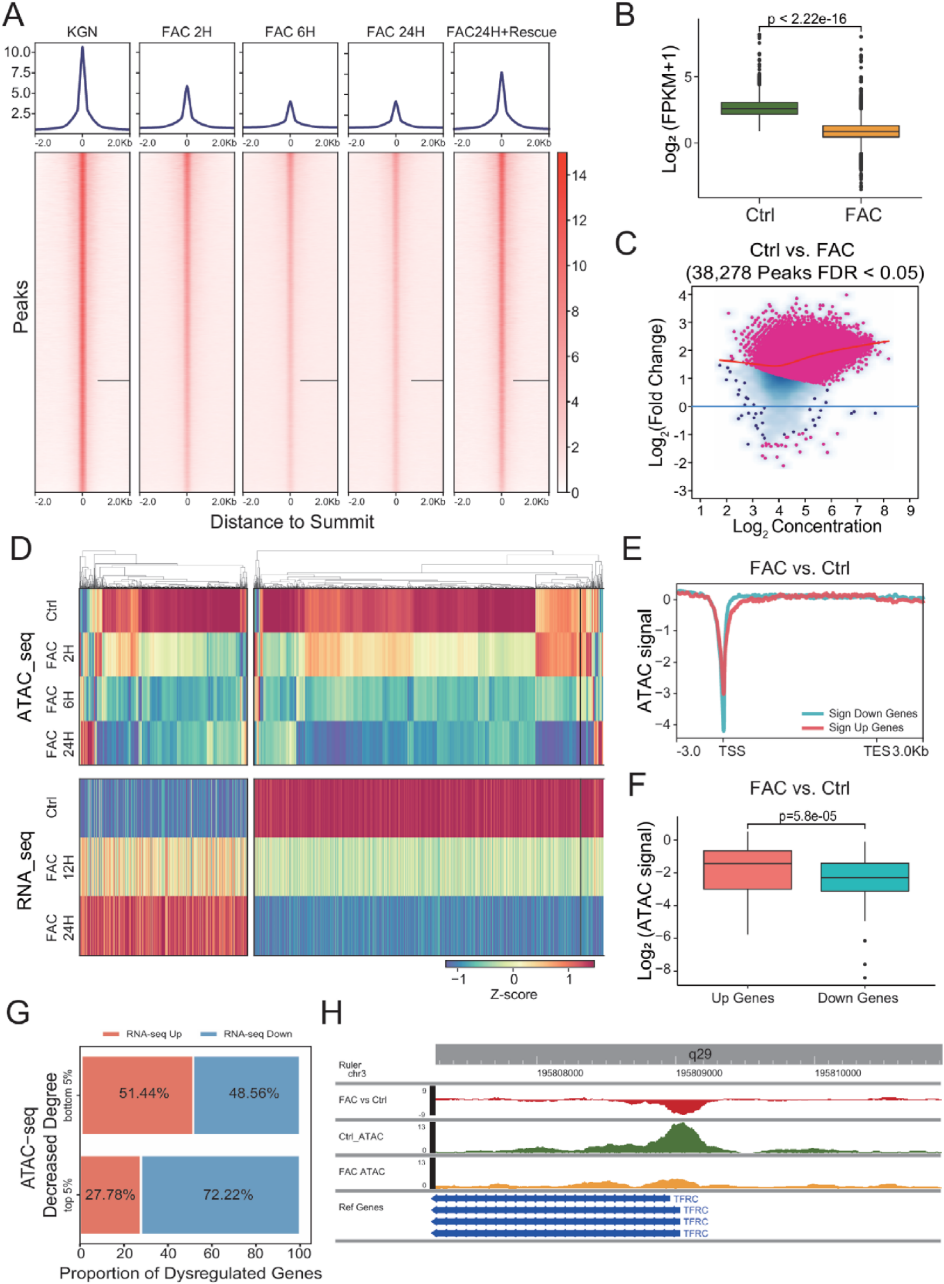
Genome‐wide decrease of chromatin accessibility was associated with transcriptional reprogramming under iron‐overload. (A) Heatmap showing ATAC‐seq signal (observed/expected calculated by MACS2) surrounding peaks of control cells after different treatments of FAC and rescue condition. (B) Quantitative comparison of ATAC‐seq reads density in peaks of control cells. Data are presented as mean ± SD (n = 42 741 peaks per condition). Statistical significance was determined by an unpaired two‐tailed Student's *t*‐test. (C) MA plot for the differential ATAC peaks after FAC treatment identified by diffbind. (D) Hierarchical clustering heatmap displaying the temporal dynamics of expression profiles of DEGs and the corresponding changes in chromatin accessibility within 1 kb around their TSS after iron treatment. (E) ATAC‐seq signal changes after FAC treatment surrounding up‐ and down‐regulated genes. (F) Quantitative comparison of ATAC‐seq signal changes within 1 kb around the TSS of DEGs after iron treatment. Data are presented as mean ± SD for upregulated genes (n = 678) and downregulated genes (n = 1310). Statistical significance was determined by an unpaired two‐tailed Student's *t*‐test. (G) Proportion of up‐ and down‐regulated genes that are nearest to the ATAC‐seq peaks exhibiting the top 5% and bottom 5% decrease after FAC treatment. (H) ATAC‐seq profile near the promoter of TFRC before and after iron‐overload.

Electron microscopy revealed localized chromatin aggregation following FAC treatment (Figure 2A), indicating higher‐order structural compaction. However, immunofluorescence showed no significant changes in heterochromatin marks H3K27me3 or H3K9me2/3 (Figure  S3A), suggesting an alternative regulatory mechanism. To further investigate the relationship between chromatin structural remodeling and transcriptional changes under iron‐overload stress, we performed ATAC‐seq to assess chromatin accessibility (Figure S3B, C, and Table S1). ATAC‐seq demonstrated a profound, genome‐wide reduction in chromatin accessibility upon iron overload. While control cells exhibited 42 741 accessible regions, only 5626 remained open after FAC treatment. Nearly all (96.7%) open regions in the FAC group overlapped with those in the control group, indicating widespread chromatin closure. Concordantly, ATAC‐seq read density at accessible sites was markedly reduced (*p* < 2.22e‐16, *t*‐test, Figure 3A,B), and among 38 278 identified differential accessible regions (DARs), 38 254 showed decreased accessibility vs. only 24 increased (Figure 3C). These results were highly reproducible across replicates (Figure  S3D–F). Strikingly, time‐course ATAC‐seq (2, 6, 24 h post‐FAC) revealed progressive loss of accessibility: 24 905 and 6113 peaks at 2 and 6 h, respectively, with 91.3% and 97.8% of these peaks overlapping control regions, highlighting a stepwise chromatin compaction (Figure 3A,D). To further test reversibility, we withdrew FAC after 24 h of treatment and cultured cells in normal medium for another 24 h. ATAC‐seq revealed extensive restoration of chromatin accessibility (Figure 3A; Figure  S3G), confirming that iron‐induced, time‐dependent chromatin closure is reversible.

Notably, chromatin closure is associated with but occurs before changes in the transcriptome. While global accessibility decreased at promoters of all the dysregulated genes, the decrease was more pronounced for the down‐regulated genes (Figure 3E). Quantitative comparison showed significantly greater loss of ATAC‐seq read density within a 1 kb region around gene transcription start sites (TSSs) of downregulated genes than upregulated genes (*p* = 5.8e‐05, Wilcoxon test, Figure 3F). Furthermore, transcriptional polarity directly correlates with chromatin accessibility gradients under iron overload. Genes nearest the top 5% most‐closed DARs were predominantly downregulated, while those near the least‐closed (bottom 5%) were enriched for upregulated genes (Figure 3G). Integrative analysis confirmed marked accessibility loss at promoters of strongly repressed genes (e.g., *TFRC*), but relative preservation near induced genes (e.g., *ZNF185*) (Figure 3H; Figure  S3H). In addition, accessibility at TSS of differentially expressed genes (DEGs) declined rapidly at 2–6 h post FAC treatment and almost plateaued by 6 h, whereas transcriptome continued to change between 12 and 24 h (Figure 3D). The temporal dynamics of normalized signals of RNA‐seq and ATAC‐seq (scaled from 0 to 1), as well as the magnitude of signal changes across consecutive time intervals, also showed that chromatin accessibility changes precede transcriptomic changes (Figure S3I,J).

Collectively, these data establish that iron overload triggers rapid, reversible, and genome‐wide chromatin compaction that preferentially silences transcriptionally downregulated loci, suggesting chromatin accessibility may contribute to iron‐mediated transcriptional reprogramming.

### 2.4 SOX4 Mediates Iron‐Responsive Chromatin Accessibility Changes in a Dose‐Dependent Manner

**FIGURE 4:**
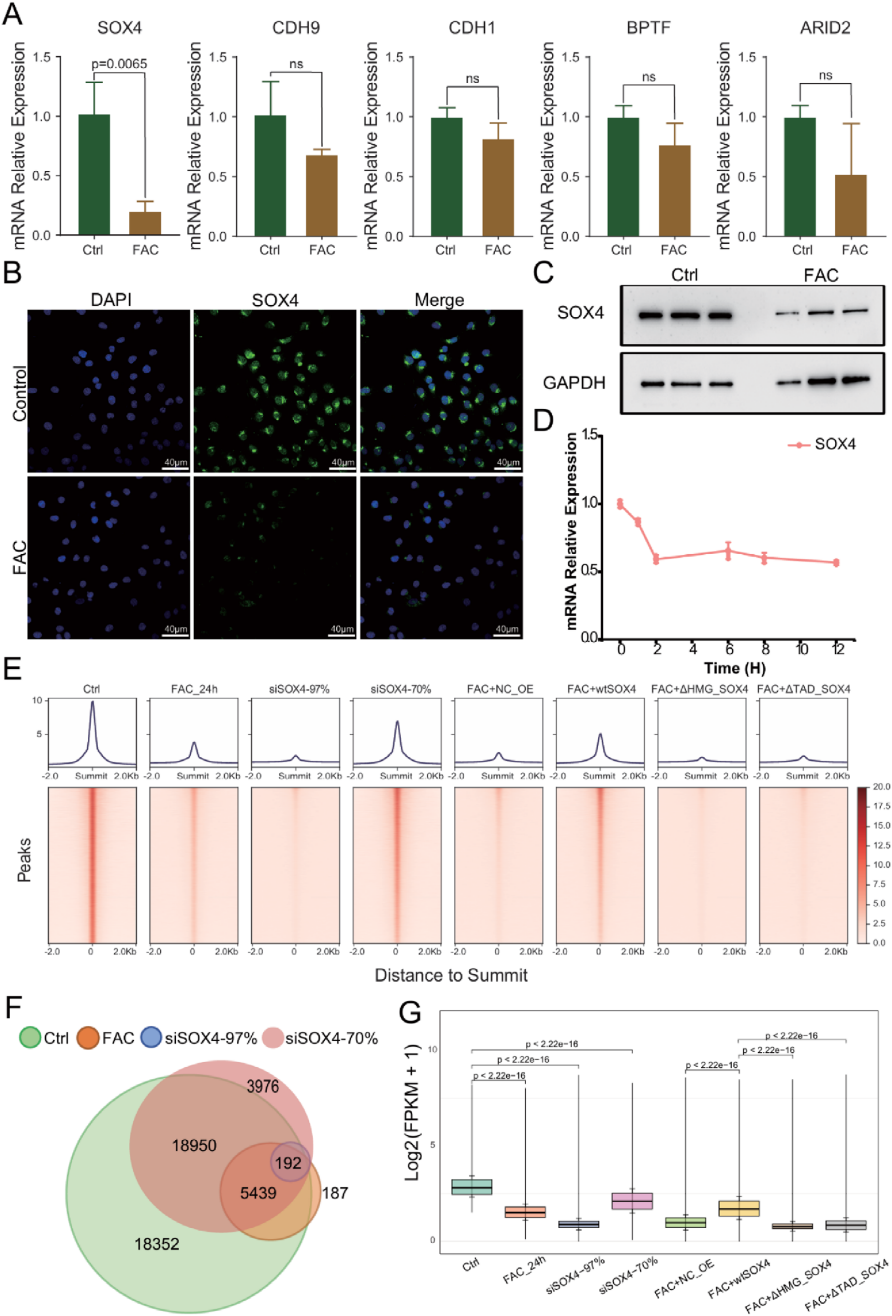
SOX4 plays core roles in regulating chromatin accessibility under iron‐overload stress. (A) qPCR validation of SOX4 and chromatin‐remodeling complex subunits identified by RNA‐seq. Data from n = 3 biological replicates are shown as mean±SD; *p* = 0.0065 for SOX4 vs. control (unpaired two‐tailed *t*‐test). Statistical notation used throughout the figure: ns, not significant; ^*^ *p* < 0.05; ^**^ *p* < 0.01; ^***^ *p* < 0.001. (B) Immunofluorescence analysis of SOX4 upon FAC treatment. (C) Western blot analysis of SOX4 upon FAC treatment. (D) Dynamic changes of SOX4 mRNA expression during FAC treatment were assessed by qPCR. Data represent mean ± SD from biological replicates (n = 3) at each time point. (E) Heatmap showing ATAC‐seq signal (observed/expected calculated by MACS2) surrounding peaks of control cells (peaks = 42 741) under different conditions: control, iron treatment, and SOX4 knockdown; as well as iron treatment combined with transfection of an empty vector (NC_OE), wild‐type SOX4 (wtSOX4), or mutant SOX4 (ΔHMG_SOX4 and ΔTAD_SOX4) overexpression plasmids. (F) Venn diagram showing the overlap of ATAC‐seq peaks after SOX4 knockdown. (G) Quantification of ATAC‐seq read density in peaks under conditions as in (E). Data are presented as mean ± SD (n = 42 741 peaks per condition); statistical significance was determined by unpaired two‐tailed Student's *t*‐test.

To identify drivers of iron overload–induced chromatin remodeling, we examined differentially expressed chromatin regulators from RNA‐seq data. qRT‐PCR analysis revealed no significant alterations in transcriptional levels of chromatin remodeling genes *CHD1*, *CHD9*, *BPTF*, and *ARID2* following FAC treatment (Figure 4A). Notably, SOX4, a lineage‐specific marker of the ovarian lineage annotated in the PaGenBase database, emerged as one of the top downregulated genes under iron overload conditions (Figure 1E) and in granulosa cells derived from endometriosis patients (Figure S4A). Given the established role of SOX family transcription factors in chromatin accessibility regulation, we validated SOX4 suppression by qRT‐PCR, western blotting, and immunofluorescence under FAC treatment (all *p* < 0.05, Figure 4B,C). Importantly, SOX4 exhibited rapid transcriptional responsiveness to iron overload, with expression kinetics paralleling chromatin accessibility dynamics (Figure 4D).

To test causality, we performed siRNA‐mediated SOX4 knockdown followed by ATAC‐seq. Strikingly, chromatin accessibility decreased in a SOX4 dose‐dependent manner: 70% knockdown (KD 70%) retained 27,713 accessible peaks, whereas 97% knockdown (KD 97%) left only 192 peaks (Figure 4E,F; Figure S4B). Nearly all KD 97% peaks (99%) overlapped with KD 70% regions, with negligible *de novo* peak formation in both conditions. This regulatory continuum paralleled the above observations: FAC‐induced about 80% SOX4 suppression (Figure 4A) produced chromatin accessibility reductions intermediate between the KD 97% and KD 70% thresholds (Figure 4G). Notably, the reversible nature of iron overload effects was evidenced by synchronized restoration of both chromatin accessibility (Figure 3A) and SOX4 expression (Figure S4C) upon the depletion of high iron stress. These findings establish a dosage dependency wherein chromatin accessibility modulation directly scales with SOX4 expression levels. As exemplified by *TFRC* gene regions, several accessible sites displayed SOX4 dose‐dependent alterations (Figure S4D). Additionally, to directly support that SOX4 acts as a sequence‐specific director of chromatin remodeling, we performed chromatin accessibility rescue experiments in FAC‐treated KGN cells by re‐expressing either wild‐type SOX4, a DNA‐binding domain (HMG) mutant, or a transactivation domain (TAD) mutant. Strikingly, re‐expression of wild‐type SOX4 robustly restored global chromatin accessibility, increasing the number of accessible regions from 179 (empty vector control) to 12 416, a ∼68‐fold enhancement. In contrast, neither the HMG DNA‐binding mutant nor the TAD mutant was capable of rescuing accessibility, yielding only 228 and 163 accessible regions, respectively (Figure 4E). Quantitative comparison further demonstrates that the rescue capacity of wild‐type SOX4 is significantly superior to that of either mutant (Figure 4G). Moreover, SOX4 downregulation under iron stress was not cell‐type specific: neural progenitor cells (NPCs), which express high basal SOX4, also exhibited reduced SOX4 upon FAC treatment (Figure S4E), underscoring its broad role in iron‐responsive gene regulation. These results demonstrate that both DNA‐binding and transactivation domains of SOX4 are important for its role in remodeling chromatin accessibility under iron‐stress conditions.

Collectively, these findings established SOX4 as a central effector governing iron‐responsive chromatin topology, with its expression dynamics dictating the magnitude of accessibility reprogramming.

### 2.5 TFEB Mediate SOX4 Repression under Iron Overload, Disrupting SOX4‐SWI/SNF Mediated Chromatin Accessibility

**FIGURE 5:**
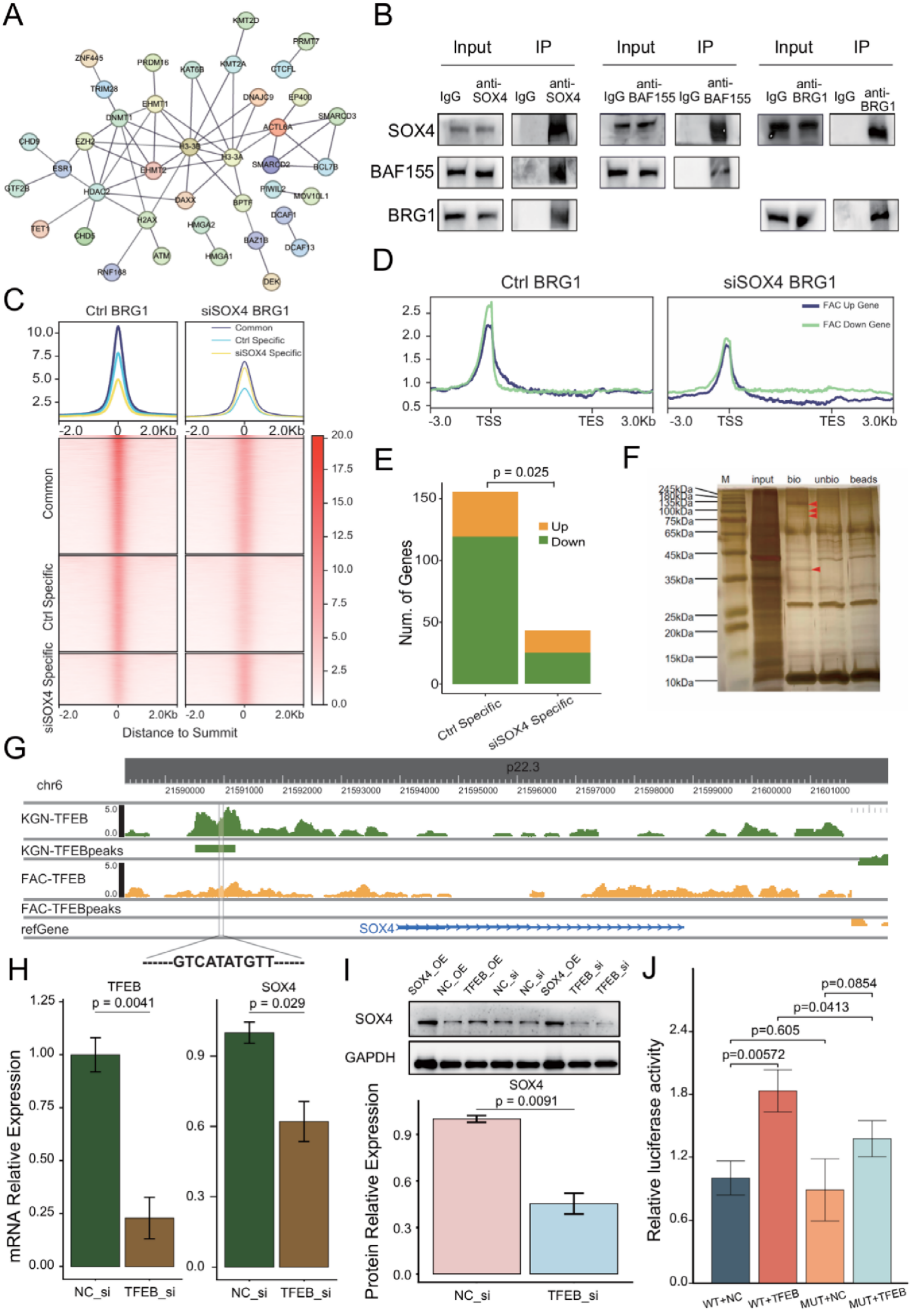
SOX4 cooperates with SWI/SNF complex to regulate chromatin accessibility. (A) Protein‐protein interaction network for proteins identified by SOX4 IP‐MS. (B) Co‐IP showing the direct interaction of SOX4 with BAF155 and BRG1 in KGN cells. (C) Heatmap illustrating BRG1 binding intensity in common and condition‐specific peaks for control and SOX4‐knockdown KGN cells. The intensity of the BRG1 signal is represented by a color gradient, ranging from low (white) to high (red). (D) Changes of BRG1 binding profile in up‐ and down‐regulated genes after SOX4 knockdown. (E) Number of up‐ and down‐regulated genes that are targeted by condition‐specific BRG1 binding peaks. Statistical significance was determined by Chi‐square test (*p* = 0.025; Ctrl specific DEGs: n = 155; siSOX4 specific DEGs: n = 43). (F) DNA pull‐down assays were used to identify proteins that bind to the SOX4 promoter. Red arrows indicate protein bands specific to the biotin group. (G) TFEB binding profile surrounding *SOX4* gene before and after FAC treatment. The CLEAR motif located within the identified binding peaks in control cells was labeled. (H) qPCR validation of SOX4 and TFEB mRNA expression following TFEB knockdown. NC_si: negative control siRNA; TFEB_si: TFEB siRNA. (I) Western blot validation of SOX4 protein expression after TFEB knockdown. OE: over‐expression. SOX4_OE was used as a positive control. (J) The activation of wild‐type (WT) and CLEAR‐mutated (MUT) SOX4 promoters by TFEB was assessed using a luciferase reporter assay. qPCR, western blot, and luciferase reporter assay were repeated three times, and the results are presented as means ± SD. Statistical significance was assessed using an unpaired two‐tailed Student's *t*‐test; exact *p* values are indicated in the figures.

To elucidate the mechanistic basis of SOX4‐mediated chromatin accessibility, we performed SOX4 immunoprecipitation‐coupled mass spectrometry (IP‐MS) and identified five SWI/SNF complex components (ACTL6A, SMARCD2, SMARCD3, BCL7B, BCL11A) (Figure 5A). Reciprocal co‐IP assays confirmed direct interaction between SOX4 and BRG1 (SMARCA4) and BAF155 (SMARCC1), core sub‐units of the SWI/SNF complex (Figure 5B), establishing that SOX4 recruits the SWI/SNF remodeling complex to modulate chromatin accessibility. We performed BRG1 ChIP‐seq to demonstrate the impact of high‐iron on SWI/SNF genomic binding, and results revealed that 83% of its binding sites overlapped with ATAC‐seq peaks, preferentially centered at accessible region summits (Figure S5B), aligning with SWI/SNF's role in maintaining chromatin accessibility. Critically, despite unchanged BRG1 levels upon SOX4 knockdown (Figure S5A), SOX4 knockdown impaired SWI/SNF DNA binding (Figure 5C), resulting in the loss of approximately 5900 BRG1 peaks and a concomitant reduction in signal intensity at the remaining binding sites. Notably, lost peaks were enriched at promoters (Figure S5C), and genes associated with lost BRG1 binding (with BRG1 peaks within ±3 kb of gene TSS) were significantly more likely to be downregulated than those with gained binding (*p* = 0.025, Chi‐square test; Figure 5E). Moreover, under FAC treatment, BRG1 binding at TSSs decreased more sharply for downregulated than for upregulated genes (Figure 5D), mirroring the ATAC‐seq dynamics. These findings suggest that SOX4 directly interacts with the SWI/SNF complex, and SOX4 suppression disrupts SWI/SNF's DNA binding, potentially driving chromatin closure and subsequent transcriptional repression in iron overload conditions.

We next sought the upstream regulator of SOX4 suppression during iron overload. A DNA pull‐down assay using the SOX4 promoter followed by MS identified TFEB, a master transcriptional regulator of lysosomal biogenesis and iron homeostasis, as a candidate binder (Figure 5F). ChIP‐seq analysis in KGN cells confirmed TFEB binding located approximately 3 kb upstream of the SOX4 TSS, with a high‐confidence CLEAR motif sequence. Notably, this binding was significantly reduced under iron overload (Figure 5G). To further clarify TFEB's regulatory role in SOX4 expression, we performed both loss‐ and gain‐of‐function experiments. SiRNA‐mediated TFEB knockdown significantly reduced SOX4 mRNA and protein levels (*p* = 0.029 and *p* = 0.0091, respectively; two‐tailed t‐test), indicating that TFEB acts as a positive regulator of SOX4 (Figure 5H,I). In overexpression studies, high‐dose TFEB transfection induced cytotoxicity, likely due to hyperactivation of autophagy, so we titrated plasmid amounts and identified a subtoxic dose that yielded a modest, though not statistically significant, increase in SOX4 expression (*p* = 0.053 by qPCR; *p* = 0.13 by Western blot, Figure S5D,E). Consistently, a dual‐luciferase reporter assay showed co‐transfection of SOX4 promoter containing the CLEAR motif with a TFEB plasmid significantly activated the reporter activity (*p* = 0.0057, *t*‐test), while mutation of the CLEAR motif significantly attenuated the level of activation (*p* = 0.041, *t*‐test, Figure 5J). These results indicate that TFEB acts as a direct positive regulator of SOX4. Taken together, our findings support a model in which iron overload reduces SOX4 levels by decreasing TFEB binding at its promoter, which in turn affects SOX4 interaction with SWI/SNF, leading to decreased chromatin accessibility and reduced gene transcription.

### 2.6 Iron Overload Preserves Large‐Scale 3D Genome Architecture Despite Reduced CTCF Binding

**FIGURE 6:**
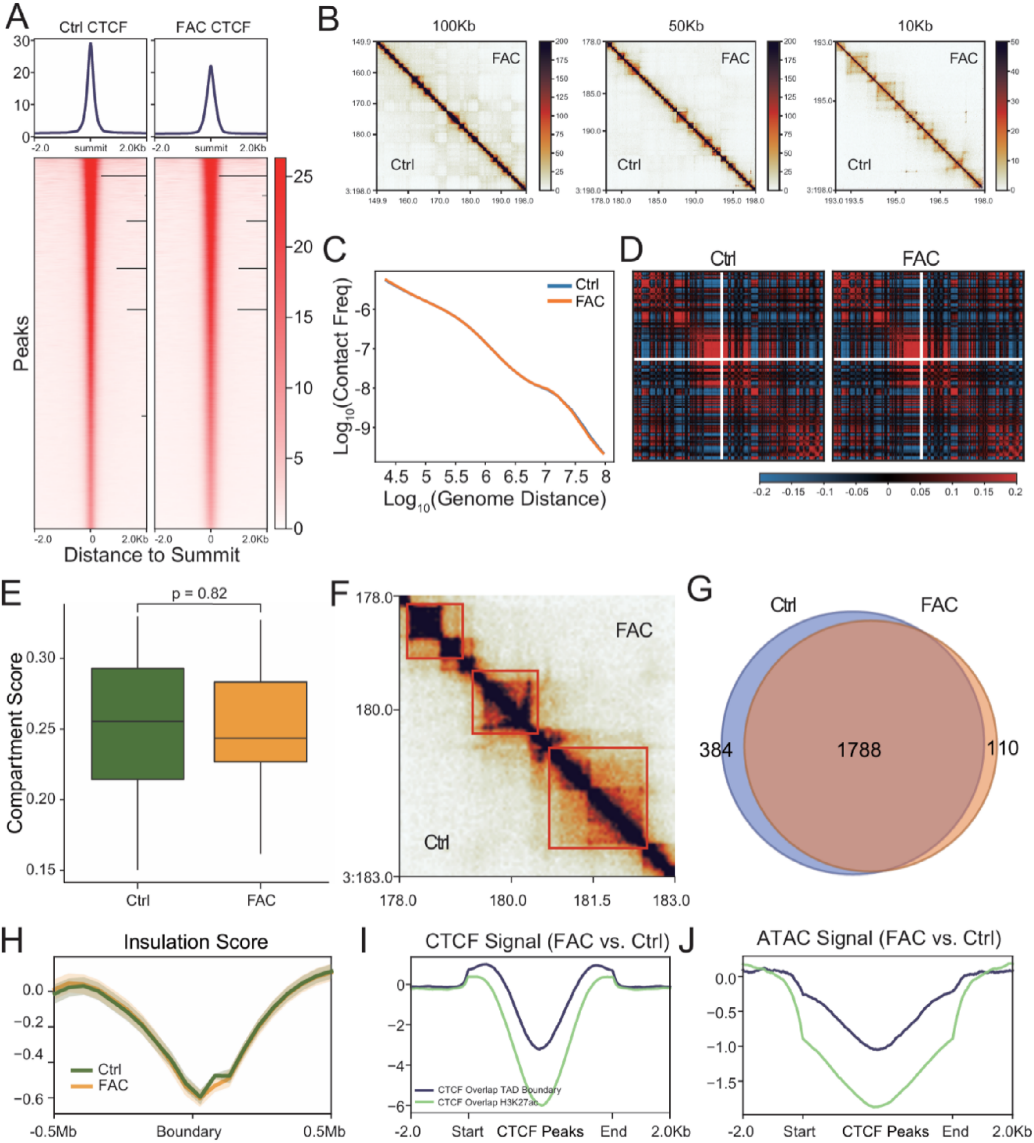
High iron does not impact large‐scale 3D genome conformation. (A) Heatmap illustrating CTCF binding intensity before and after FAC treatment in KGN cells. (B) Hi‐C contact matrix of chromosome 3 at 100 kb, 50 kb, and 10 kb resolution compared between control (lower left) and FAC‐treated (upper right) cells. (C) Contact frequency decay curves at control and FAC‐treated conditions. (D) Chromatin compartmentalization at the two conditions. Chromosome 3 is shown as an example, with the first eigenvector profiles displayed; compartment B is colored blue and compartment A orange. (E) Genome‐wide compartment scores at each condition (*p* = 0.82, paired two‐tailed Student's *t*‐test). (F) TADs detected in a 5 Mb region of chr3 were shown as an example, together with the corresponding contact maps. (G) Venn diagram showing the overlap of identified TADs in different conditions. Shared TADs were defined as overlapping regions that cover more than 80% of each corresponding TAD. (H) Genome‐wide insulation score profiles around TAD boundaries at the two conditions. Statistical significance was determined by unpaired two‐tailed Student's *t*‐test (*p* = 0.99; n=3938 and 3859 boundaries for Ctrl and FAC groups, respectively). (I) Changes of CTCF binding profile in structural (overlapping TAD boundaries) and functional (overlapping H3K27ac peaks) CTCF sites after FAC treatment. (J) Changes of ATAC‐seq signal in structural (overlapping TAD boundaries) and functional (overlapping H3K27ac peaks) CTCF sites after FAC treatment.

The global decline in chromatin accessibility triggered by high‐iron stress prompted us to explore its impact on 3D genome structure remodeling. Although CTCF occupancy was overall reduced (Figure 6A), Hi‐C analysis revealed remarkable preservation of large‐scale 3D genome structure. Biological replicates showed high reproducibility at 100 kb resolution (Median GenomeDISCO scores: 0.951 for control and 0.950 for FAC treatment; Figure S6A), with ∼300 million valid read pairs per condition (Table S2). Contact matrices were highly similar across resolutions (Figure 6B), and contact frequency decay curves exhibited minimal divergence (Jensen–Shannon divergence = 3.77e‐05; Figure 6C). The inter‐group GenomeDISCO score (0.941) further confirmed overall structural stability. Additionally, compartmentalization was also unaffected both in position and strength (Figure 6D). Only 4% of the genome was undergoing A/B compartment switching (Figure S6B), and median compartment scores (AB/AA+BB) were nearly identical (control: 0.255; FAC: 0.243; *p* = 0.82, paired *t*‐test; Figure 6E). Saddle plots confirmed comparable A‐B interaction depletion (Figure S6C), and H3K27ac enrichment, a hallmark of active (A) compartments, remained stable after FAC treatment (Figure S6D).

Moreover, topologically associating domains (TADs) were similarly conserved (Figure 6F). At 50 kb resolution, 2125 and 2060 TADs were identified in control and FAC groups, respectively, with 1788 (84%) shared (≥80% reciprocal overlap, Figure 6G). TAD boundary strength, measured by insulation scores, showed no significant difference (mean IS: ‐0.6037 vs. ‐0.6025; *p* = 0.99; Figure 6H). As expected, CTCF peaks were enriched at TAD boundaries, with higher overlap compared to that in randomly chosen regions (Figure S6E). Notably, dividing CTCF sites into structural (overlapping with TAD boundaries) and functional (overlapping with H3K27ac ChIP‐seq peaks) groups, we found that functional CTCF, which may mediate loops and regulate gene expression, was more affected than structural CTCF upon iron overload (Figure 6I), and showed more pronounced reductions in local chromatin accessibility (Figure 6J). Collectively, iron stress preserves global 3D architecture despite chromatin compaction and selective CTCF loss at regulatory sites, implicating loop reorganization in transcriptional reprogramming.

### 2.7 High Iron Stress Regulates Iron‐Responsive Gene Transcription by Inhibiting Chromatin Loop Formation

**FIGURE 7:**
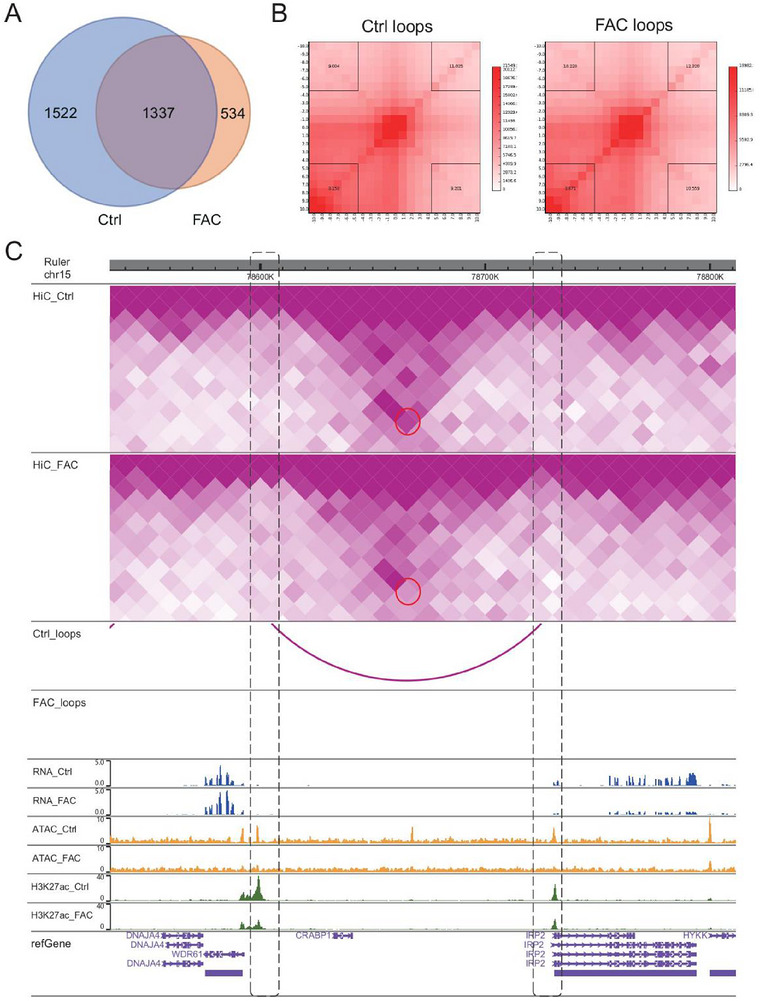
High iron stress regulated iron‐responsive gene transcription by inhibiting the formation of chromatin loops. (A) Number and proportion of chromatin loops overlapping between the two conditions. (B) Aggregate loop plots showing the strength of interactions between loop anchors. (C) Schematic of the Hi‐C contact map, along with annotated chromatin loops, RNA‐seq, ATAC‐seq, and H3K27ac ChIP‐seq profiles in the genomic region surrounding the *IRP2* gene. A control‐specific chromatin loop connects the *IRP2* promoter to a distal enhancer. Upon FAC treatment, both loop anchors exhibit reduced H3K27ac enrichment and decreased ATAC‐seq signal, concomitant with downregulation of *IRP2* expression.

Using HiCCUPS at 10‐kb resolution, we identified 2859 high‐confidence chromatin loops (from 5352 anchors) in control cells and 1,868 loops (from 3617 anchors) after FAC treatment, with 1337 loops conserved between conditions, defined as those with both anchors shifted no more than one bin (Figure 7A). Aggregate peak analysis (APA) confirmed strong Hi‐C signal enrichment at predicted loop anchors (Figure 7B). In control cells, 58% of loops had CTCF bound at both anchors and 30% at one anchor. Moreover, the majority of loops connected promoters to enhancers, with enhancers defined by H3K27ac ChIP‐seq (Figure S7A).

Alterations in chromatin loops have been linked to transcriptional reprogramming. Consistent with this, we found that loops linked to downregulated genes generally weakened after FAC treatment, whereas those associated with upregulated genes showed a trend toward strengthening (though not significantly; Figure S7B). Similar trend was also observed by Activity by Contact (ABC) model, which sensitively links regulatory elements to their target genes through a combination of ATAC‐seq, H3K27ac ChIP‐seq data as well as Hi‐C data. Results revealed that the number of predicted enhancers associated with downregulated genes decreased significantly, whereas those linked to upregulated genes remained largely unchanged (*p* = 0.0055, *t*‐test, Figure S7C). Furthermore, among genes that lost enhancers, a significantly higher proportion were downregulated (70.1%) compared to those that gained enhancers (61.4%, *p* = 0.0073, Chi‐squared test, Figure S7D). To further identify loop‐regulated targets, we intersected differentially expressed genes (DEGs) with condition‐specific loops and identified 23 upregulated genes connected by FAC‐specific loops and 126 downregulated genes associated with control‐specific loops (Tables S3 and S4). These genes were significantly enriched in the ‘inorganic ion homeostasis’ pathway (Figure S7E), implicating that chromatin loop remodeling contributes to iron homeostatic regulation under high‐iron stress. Notably, this set included *IRP2* and *TFRC*, two core regulators of iron metabolism. Analyzing chromatin changes around these genes, we observed coordinated epigenetic modifications regulating their expression under FAC treatment (Figure 7C; Figure S7F). At the *IRP2* locus, a control‐specific loop connecting its promoter was lost after FAC treatment, accompanied by reduced promoter accessibility, diminished H3K27ac, and decreased looping strength to the distal enhancer (Figure 7C). Similarly, a CTCF‐anchored loop at *TFRC* showed decreased CTCF binding at both anchors and reduced looping strength (Figure S7F). Together, these results demonstrate that fine‐scale loop structure and local chromatin accessibility jointly orchestrate the transcriptional response of granulosa cells to iron‐overload stress.

### 2.8 SOX4 Serves as a Central Hub for Chromatin Accessibility Reprogramming in Granulosa Cells Across Distinct Pathological Stimuli

**FIGURE 8:**
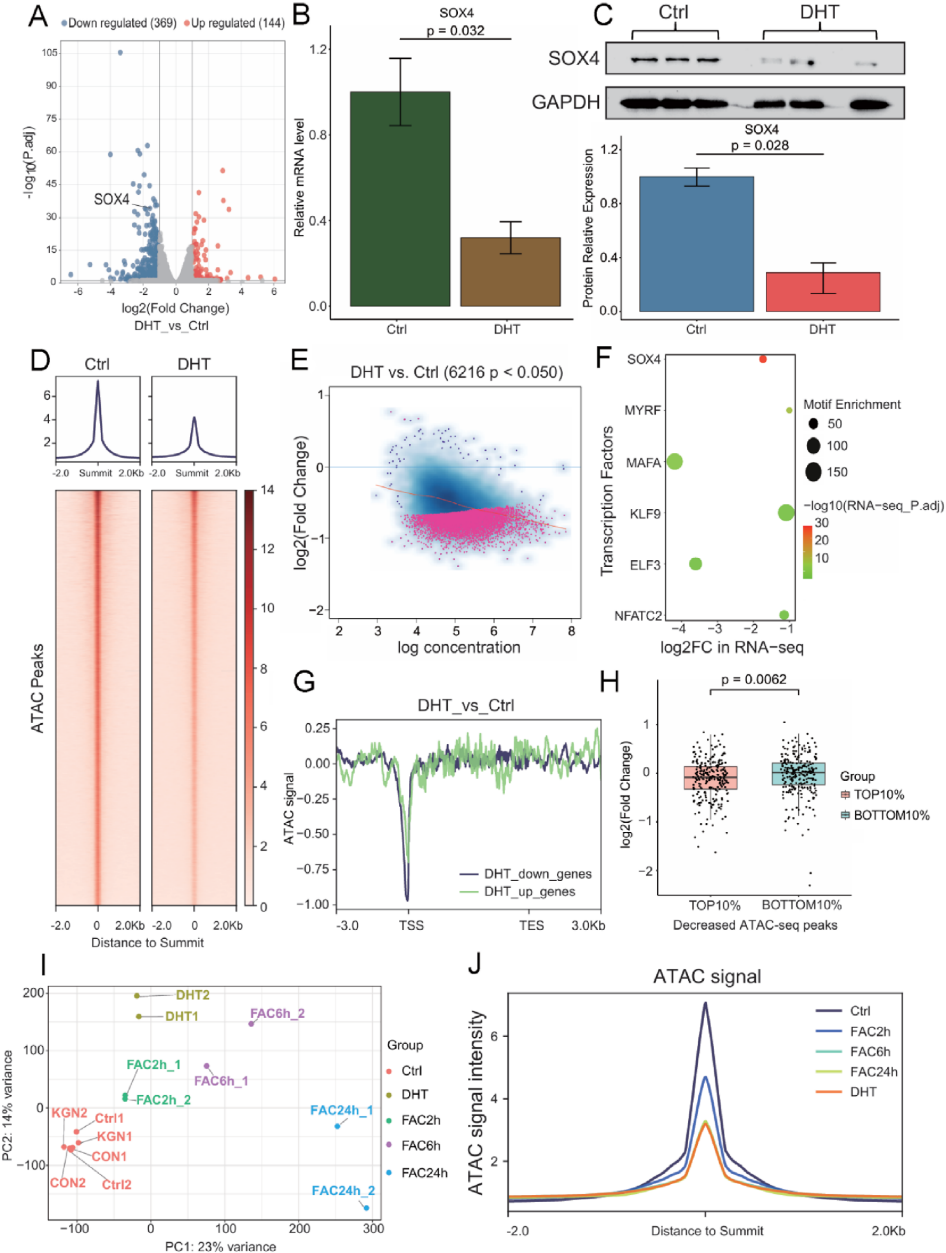
SOX4‐dependent chromatin accessibility remodeling under androgen stimulation. (A) Volcano plot of DEGs between control and DHT‐treated KGN cells. (B) qPCR validation of SOX4 mRNA expression following DHT treatment. (C) Western blot validation of SOX4 protein expression after DHT treatment. qPCR and WB were repeated three times, and the results are presented as means ± SD. Statistical comparisons were performed using an unpaired two‐tailed Student's *t*‐test. (D) Heatmap of ATAC‐seq signals (observed/expected) comparing DHT‐treated with control cells. (E) MA plot of the identified differential ATAC peaks after DHT treatment. (F) Intersection of transcription factors whose motifs are significantly enriched in decreased ATAC‐seq peaks and that are downregulated in RNA‐seq. The log2FC and *p* value of RNA‐seq, as well as ‐log(*p*‐values) of motif enrichment, were shown. (G) Changes of ATAC‐seq signals near upregulated and downregulated genes before and after DHT treatment. (H) Log_2_fold change (Log_2_FC) of up‐ and down‐regulated genes that are nearest to the ATAC‐seq peaks exhibiting the top 10% and bottom 10% decrease after DHT treatment. Data are presented as mean ± SD (n = 253 genes per condition). Statistical significance was determined by the Wilcoxon rank‐sum test. (I) PCA plot of ATAC‐seq samples under iron overload and high androgen stimulation. (J) ATAC‐seq signal profiles across different treatments after merging biological replicates.

Given the dose‐dependent role of SOX4 in orchestrating chromatin accessibility under FAC exposure, we extended our investigation to determine whether it similarly mediates responses to hyperandrogenism, a hallmark feature of polycystic ovary syndrome (PCOS). Notably, RNA‐seq revealed that SOX4 expression was markedly downregulated following androgen stimulation (25 nm DHT for 24 h), confirmed by qRT‐PCR and Western blotting (*p* = 0.032 and 0.028 for qPCR and Western blot, *t*‐test, Figure 8A–C). Concordantly, ATAC‐seq analysis demonstrated a striking global decrease of chromatin accessibility (Figure 8D), with 6216 differentially accessible regions (DARs) identified ‐ 99.95% of which (6213) were closed (Figure 8E). Motif enrichment in closed DARs revealed strong overrepresentation of SOX4‐binding motifs (Figure 8F), suggesting that SOX4 depletion directly drives accessibility loss.

Gene transcriptional regulation and chromatin accessibility changes are also correlated upon DHT exposure, paralleling the pattern observed in iron overload. Downregulated genes exhibited more pronounced reductions in chromatin accessibility compared with upregulated genes (Figure 8G, *p* = 0.044, *t*‐test). In addition, genes adjacent to the most strongly decreased ATAC‐seq peaks (top 10% ranked by fold change) were preferentially downregulated, in contrast to those near the least decreased peaks (bottom 10% ranked by fold change, Figure 8H, *p* = 6.2e‐03, Wilcoxon test). Moreover, comparative analysis of iron overload and androgen stimulation revealed striking convergence. PCA showed tight replicate clustering, with FAC‐treated samples progressively shifting along PC1 over time, while DHT‐treated cells positioned between the 2 and 6 h FAC groups (Figure 8I; Figure S8A), a pattern corroborated by similar trends in ATAC‐seq signal intensity (Figure 8J). Notably, SOX4 downregulation was more pronounced after DHT stimulation than after 2 h of FAC exposure, reinforcing its dose‐dependent role in maintaining chromatin openness. Finally, overlap analysis showed that DARs induced by DHT were almost a subset of those identified under FAC 24 h exposure (Figure S8B), and shared DARs were significantly enriched for SOX4‐binding motifs (*q* < 0.05).

Collectively, these findings establish SOX4 as a central, dose‐sensitive regulator that integrates diverse stress signals, such as iron excess and hyperandrogenism, to orchestrate chromatin accessibility and transcriptional adaptation in granulosa cells.

## 3 Discussion

**FIGURE 9:**
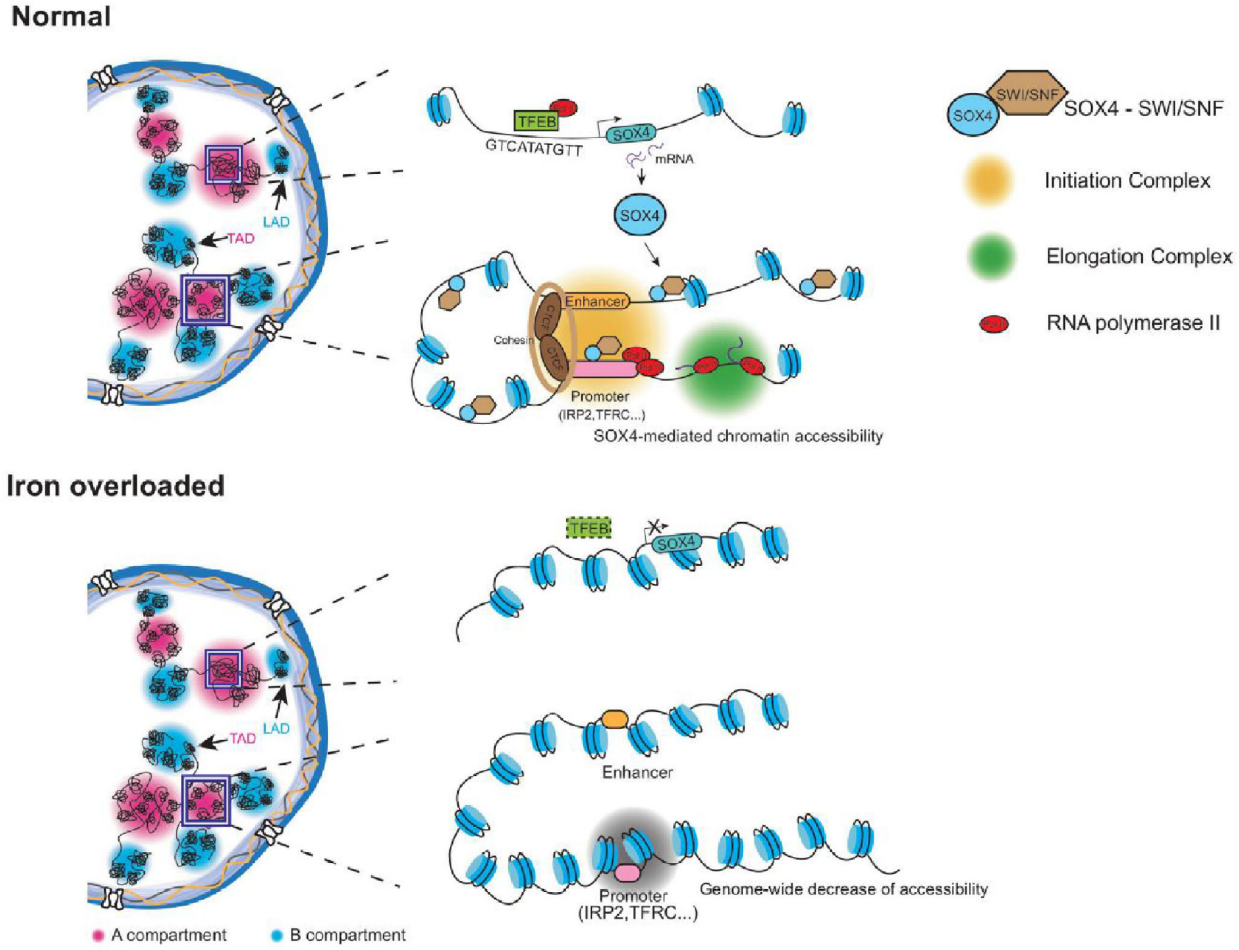
Schematic diagram showing the main discovery of this study.

The cell stress model emerges as a superior framework for exploring the intricate interplay between transcriptional reprogramming and chromatin remodeling. Iron overload, which is a pathophysiological condition implicated in numerous human disorders, triggers cellular stress and disrupts cellular homeostasis. In this study, we systematically explored cellular responses to iron excess by integrating transcriptomic, epigenomic, and chromatin conformation analyses in KGN cells. Notably, the transcriptional profiles of KGN cells exhibited significant changes akin to those observed in clinical samples. Our findings reveal that iron overload induces a global reduction in chromatin accessibility, which correlates with widespread transcriptional downregulation. While large‐scale chromatin architecture remains largely intact, fine‐scale chromatin loops undergo dynamic reorganization, coinciding with altered expression of key iron‐regulatory genes such as IRP2 and TFRC. Mechanistically, SOX4 is established as a dosage‐sensitive master regulator of chromatin accessibility. Under iron stress, binding of TFEB to the CLEAR motif in the SOX4 promoter is attenuated, leading to transcriptional downregulation of SOX4. The consequent reduction in SOX4 levels impairs its functional interaction with the SWI/SNF chromatin remodeling complex, ultimately resulting in diminished chromatin accessibility (Figure 9). Notably, similar to iron overload, hyperandrogenism phenocopied the downregulation of SOX4 and its associated reduction in chromatin accessibility, suggesting a conserved regulatory axis. Together, our findings provide a comprehensive molecular framework for understanding epigenetic reprogramming under iron stress, highlighting the pivotal role of the TFEB‐SOX4‐SWI/SNF axis in mediating cellular adaptation to high‐iron environments, beyond the canonical IRE‐IRP regulatory pathway.

The spatiotemporal hierarchy of chromatin accessibility dynamics and 3D genome organization constitutes a fundamental regulatory axis in eukaryotic stress adaptation. Our findings establish that iron overload induces dynamic chromatin accessibility remodeling coupled with fine‐scale loop restructuring, while preserving macroscale chromatin topology. This architectural resilience mirrors observations under heat shock [13, 20], where global 3D structure maintenance accompanies transcriptional reprogramming, but contrasts sharply with hyperosmotic stress‐induced transient chromatin decondensation [10]. Notably, the observed iron‐responsive accessibility changes precede transcriptional alterations, recapitulating hypoxia response kinetics [11], yet diverging from heat shock's immediate transcriptional activation prior to chromatin reconfiguration. Additionally, in response to hormones, the borders of topologically associating domains (TADs) predominantly persist, whereas interactions within TADs exhibit correlations with alterations in gene expression [21]. These observations suggest that mammalian cells might employ diverse regulatory strategies and respond heterogeneously to orchestrate transient fluctuations in gene expression when confronted with a multitude of stimuli. Mechanistically, the intricate stimulus‐specific architectural hierarchy within the genome offers profound insights into the differential engagement of chromatin remodelers in various cellular processes, such as SWI/SNF‐mediated accessibility modulation (as shown here) and cohesin‐driven loop reorganization. This plasticity aligns with the emerging paradigm of context‐dependent topological selectivity, where core scaffolding elements (TAD boundaries, compartments) maintain structural integrity while regulatory loops undergo stimulus‐specific reconfiguration. Our identification of functional CTCF site sensitivity to iron overload further supports a model wherein architectural proteins serve dual roles: maintaining structural resilience while dynamically participating in stress‐responsive transcriptional regulation through allosteric chromatin interactions.

The mechanism underlying chromatin accessibility has garnered extensive attention, with emerging evidence indicating that the homeostatic maintenance of accessibility is dynamically regulated through a competitive interplay between chromatin‐binding factors and nucleosomes [22]. Among the chromatin‐binding protein, the pioneer factors, such as FoxA and GATA proteins, possess the unique ability to open closed chromatin regions [23]. Studies have indicated that these pioneer factors possess the capability to recruit nucleosome‐remodeling complexes as well as chromatin‐modifying enzymes. Such recruitment facilitates the restructuring of silent chromatin, the addition of active chromatin modifications, and ultimately, the activation of genes [24]. SOX4 belongs to the SOX family, several members of which, including SOX2, SOX9, and SOX11, have been established as pioneer transcription factors. Our data demonstrate that SOX4 modulates chromatin accessibility under iron overload by recruiting the SWI/SNF remodeling complex, raising the possibility that SOX4 may function as a pioneer factor in this context. This hypothesis is supported by a recent study, which revealed that SOX4 can induce hepatobiliary metaplasia in the adult mouse liver via its pioneer factor—mediated activity [25]. Notably, our study is the first to demonstrate the critical role of SOX4 pioneer‐factor activity in the cellular response to iron stress and to reveal its central function in stress‐induced epigenomic reprogramming. Furthermore, SOX4, recognized as a hallmark gene of the ovary, plays an indispensable role in ovarian development. The impairment of SOX4 function and the consequent decline in chromatin accessibility, triggered by elevated iron or hyperandrogenism, underscore its pivotal role in ovarian pathophysiologies such as endometriosis and PCOS. This discovery paves the way for novel therapeutic strategies targeting these metabolic stressors in infertility.

Transcription factor EB (TFEB) stands as a key regulator of cellular adaptation response to a myriad of stressors, primarily through its integral involvement in the lysosomal/autophagy pathway. Beyond its canonical function, TFEB also assumes a critical role in the fine‐tuning of cellular iron metabolism, a function underscored by the lysosome's status as a vital organelle for iron storage within the cell [26, 27]. Emerging evidence reported that TFEB over‐expression facilitated lysosomal iron import and increases the pool of iron‐laden peripheral lysosomes, which can prevent iron overload and mitigate iron‐mediated cytotoxicity [27, 28]. In our study, we establish the TFEB‐SOX4 regulatory axis as a key mechanistic link between iron overload and chromatin remodeling, thereby uncovering a parallel epigenetic regulatory layer operating alongside the classic IRP‐IRE system. While our findings clarify this pathway in the context of experimental iron overload, the contribution of the TFEB‐SOX4 regulatory axis to other iron overload disorders, such as chronic liver disease, cancer, and infections, remains largely unexplored. Consequently, this area warrants rigorous and in‐depth investigation in the future to unravel its full potential and clinical implications. Furthermore, a recent investigation has unveiled that iron regulatory protein 2 (IRP2) plays a supportive role in facilitating the nuclear translocation of TFEB [29]. In the context of *Irp2*‐deficient liver tissue or macrophages, a notable reduction in the nuclear localization of TFEB was observed, subsequently resulting in the downregulation of TFEB target genes. However, the existence of analogous regulatory mechanisms governing TFEB or potential cross‐talk between TFEB and IRP2 under conditions of high iron stress remains an open question, warranting further exploration in future studies.

From a therapeutic perspective, the TFEB‐SOX4 axis holds significant promise, particularly in stress‐affected granulosa cells under pathological conditions such as endometriosis and polycystic ovary syndrome. Under stresses like iron overload or hyperandrogenism, controlled activation of SOX4, for example, through specific agonists, could help restore appropriate transcriptional and epigenetic responses, potentially mitigating disease progression. Furthermore, pharmacological activation of TFEB offers a parallel strategy, especially relevant in iron‐overload disorders, where it could simultaneously address iron mislocalization and chromatin compaction. This dual mechanistic approach warrants further exploration, particularly in hematopoietic stem cells, where iron‐TFEB dynamics critically regulate differentiation [30]. Conversely, in conditions where SOX4 is pathologically overexpressed (e.g., in certain cancers) [31], our findings suggest that modulating cellular iron homeostasis could be leveraged to suppress the TFEB‐SOX4 axis, thereby curbing SOX4‐driven pathogenesis. More directly, targeted inhibition of SOX4 itself or its interaction with the SWI/SNF complex may offer a precise strategy to disrupt the downstream chromatin remodeling events that fuel disease progression. These not only underscore the translational significance of this iron‐sensitive epigenetic pathway but also delineates a concrete and promising direction for translating these mechanistic insights into clinically relevant interventions, thereby expanding the scope and impact of the present study.

There are some limitations of our study. First, the reliance on the KGN cell line model, although informative, necessitates validation in vivo. Future work using mouse models of endometriosis or iron overload would help translate these mechanistic findings into physiological contexts. Considering limited granulosa cells from mouse ovaries, single‐cell genomics, such as scRNA‐seq and scATAC‐seq, offer promising approaches to examine chromatin and transcriptional dynamics in native granulosa cells under disease‐relevant conditions. Second, while our time‐course data show that chromatin compaction occurs prior to transcriptome alterations, our RNA‐seq captures steady‐state mRNA levels. Since steady‐state RNA reflects not only nascent transcripts but also post‐transcriptional regulation (e.g., RNA stability, miRNA activity), which can obscure a direct causal inference between chromatin accessibility and transcription. Therefore, direct measurements of nascent RNA are needed to clarify whether accessibility changes actively block Pol II recruitment and drive transcription reprogramming, a key question for understanding the causal relationship between epigenetic and transcriptional remodeling under iron overload.

In conclusion, the realm of chromatin structure remodeling exploration and its far‐reaching implications has emerged as a highly productive and rapidly evolving field of research. Over the course of its development, investigations into chromatin accessibility remodeling and 3D genome organization have unearthed profound revelations, casting light on the intricate mechanisms underlying fundamental epigenetic reprogramming. In this study, we provide a comprehensive analysis of the regulome associated with iron overload, integrating chromatin accessibility, chromatin structure, and transcriptomic information. Our results underscore the critical role of TFEB‐SOX4 regulatory axis in chromatin remodeling, a process vital to the cellular response to iron overload and hyperandrogenism. This work not only deepens our comprehension of the complex interplay between chromatin accessibility, the three‐dimensional architecture of chromatin, transcriptional regulation, and the pathogenesis of iron overload‐related disorders but also lays the groundwork for future research endeavors aimed at unraveling the underlying molecular mechanisms and developing targeted therapeutic interventions.

## 4 Experimental Section

### 4.1 Iron Overload and Hyperandrogenism Cells Model In Vitro

The KGN cells maintain the function of steroid hormone synthesis and the characteristics of granulosa cells, and are often used to study the proliferation, apoptosis, hormone secretion, and receptor expression of granulosa cells [32, 33]. KGN cell lines were purchased from Procell System Company (CL‐0603, Wuhan, China). Cells were cultured in Dulbecco's modified Eagle's medium Ham's F‐12 (Gibco, USA) containing 10% fetal bovine serum (HyClone) and 1% penicillin/streptomycin (Gibco) (completed medium) for 18 h at 37°C in a CO_2_ incubator, as mentioned in our previous study [34]. Ferric ammonium citrate (FAC) (Sigma–Aldrich, USA) was used to induce the iron overload model [19]. To establish the in vitro hyperandrogenism stimulation model, KGN cells were seeded at a density of 2 × 10^5^ cells per well in 6‐well plates. After cell attachment, cells were serum‐starved in serum‐ and antibiotic‐free Ham's F‐12 medium for 4 h. Subsequently, cells were treated with 25 nm dihydrotestosterone (DHT, AbMole, USA) for the experimental group or with 0.1% DMSO for the control group and incubated for an additional 24 h. The FeRhoNox‐1 probes (GORYO Chemical, Japan) is an activatable fluorescent probe that specifically detects labile Fe^2+^ ions via orange (red) fluorescence. For the intracellular iron assay, ∼10^6^ cells were incubated with 5 µm of FeRhoNox‐1 probes for 30 min at 37°C in the dark. Flow cytometry was used to quantitate the intensities of fluorescence.

### 4.2 GC Isolation of Endometrioma Patients

Follicular fluid samples were collected from infertile women diagnosed with unilateral endometrioma, who underwent IVF‐ICSI procedures in the Reproductive Medicine Center of Sun Yat‐Sen Memorial Hospital. Granulosa cells were gathered with Ficoll solution (Lymphoprep; Axis‐Shield) by density gradient centrifugation. To minimize post‐aspiration cell death, all isolation procedures were completed within 1 h after follicular fluid collection. The study was approved by the medical ethics committee of Sun Yatsen Memorial Hospital, Sun Yat‐sen University (approval number: SYSEC‐KY‐KS‐2020‐143). Written informed consent was obtained from the participant.

### 4.3 Detection of Cell Cycle and Apoptosis

For the cell cycle assay, 1.0 × 10^6^ cells were harvested and suspended in 1 mL of DNA staining solution and 10 µL of permeabilization solution, incubated at room temperature in the dark for 30 min before flow cytometric analysis. For the cell apoptosis assay, 5.0 × 10^5^ cells were resuspended in 500 µL of binding buffer with 5 µL of Annexin V (Beyotime) and 5 µL of Propidium Iodide Reagent (Beyotime), incubated at room temperature in the dark for 15–20 min, and quantitated by flow cytometry.

### 4.4 RNA Sequencing (RNA‐Seq)

Total RNA was extracted from KGN cells treated with varying concentrations of FAC using the Trizol reagent (Invitrogen, USA), following the manufacturer's instructions. The quality of isolated RNA was examined by NanoDrop ND‐1000 (NanoDrop, DE, USA) and Agilent 2100 Bioanalyzer (Agilent, Santa Clara, CA). High‐quality RNA samples were then subjected to paired‐end library preparation according to the standard Illumina protocol (Illumina, San Diego, CA). The resulting libraries were sequenced on an Illumina NovaSeq sequencing platform to generate 2 × 150 bp paired‐end reads.

### 4.5 Transmission Electron Microscopy of KGN

KGN cells were seeded in 10‐cm dishes (Corning) at a density of 5 × 10^5^ cells. After treating with or without 1.5 mm FAC for 12 or 24 h, KGN cells were released by trypsin/EDTA and collected in Eppendorf tubes. The cell pellets were suspended and fixed with 2.5% glutaraldehyde (Servicebio, China) at 4°C overnight. The fixed cells were washed in 0.1 m phosphate buffer (pH 7.4) 3 times for 3 min each. A 2% low melting point agarose solution was prepared by heating and dissolving it in advance. After being cooled to about 40°C, the agarose solution was added to wrap the cell pellets. Subsequently, the cells were dehydrated with a graded series of ethanol and gradually penetrated with resin. Then, the cells were embedded in pure LR white resin (Ladd Research Industries, Burlington, VT). The ultrathin sections were cut to 70–80 nm thickness on an ultramicrotome (Leica, UC7) and mounted on 150 mesh nickel grids. The sections were subjected to double staining with uranyl acetate and lead citrate before being observed under a transmission electron microscope (Hitachi HT7800).

### 4.6 Passive Nuclear Import Experiments

The diffusion of FITC‐dextran through the nuclear envelope was tested to evaluate their passive permeability with confocal laser‐scanning microscopy. Cells were grown on glass‐bottom dishes and selectively permeabilized by incubation with 20 µg/mL digitonin in transport buffer (TB, 20 mm HEPES, 110 mm K‐Acetate, 5 mm Na‐Acetate, 2 mm Mg‐Acetate, 1 mm EGTA (pH 7.3), 2 mm DTT) supplemented with 200 µg/mL FITC‐dextran of defined molecular weights (10, 30–50 or 70 kDa; Sigma–Aldrich, Steinheim, Germany). The 70 kDa FITC‐dextran served as an integrity marker of nuclear pores and nuclear envelope. Confocal microscopy images were taken in the mid‐plane of the nuclei using a laser scanning confocal microscope (Zeiss LSM 800 with airyscan) at a rate of one image per minute. Subsequently, FITC‐dextran influx dynamics were evaluated by calculating the ratio of intranuclear fluorescence intensity to extracellular background intensity over time.

### 4.7 Immunofluorescence

Cells were fixed in 4% neutral buffered formalin for 1 h at room temperature, then washed with PBS, and resuspended in 0.1% TritonX‐100 for 15 min. Cells were washed with PBS and blocked in goat serum, then incubated with anti‐SOX4 antibody (Diagenode, Belgium) and/or anti‐Lamin B1 antibody (Abcam,United Kingdom) in blocking buffer at 4°C overnight in a humidified chamber. Goat anti‐Rabbit IgG (H+L), highly cross‐adsorbed secondary antibody Alexa Fluor 488 conjugate (Thermo Fisher, America) was used at a concentration of 4 µg/mL in PBS containing 0.2% BSA for 45 min at room temperature. DAPI was used for nuclear staining. Fluorescence images were captured by a laser scanning confocal microscope (Zeiss LSM 800 with airyscan).

### 4.8 RNA Interference

siRNA duplexes targeting *SOX4* gene (sense: 5’‐GGCACAUCAAGCGACCCAUTT‐3’, antisense: 5’‐AUGGGUCGCUUGAUGUGCCTT‐3’) and *TFEB* gene (sense: 5’‐GACGAAGGUUCAACAUCAATT‐3’, antisense: 5’‐ UUGAUGUUGAACCUUCGUCTT‐3’), along with a non‐targeting negative control siRNA, were purchased from GenePharma (Shanghai, China). KGN cells were seeded in 6‐well plates at a density of 1 x 10^6^ cells, incubated in DMEM with 10% FBS for 12 h, and then in DMEM without serum for 24 h. Transfection was subsequently conducted by siRNA (10 ug) and Lipofectamine 2000 (5uL) according to the manufacturer's protocol. After 24 h, the transfection medium was replaced with fresh DMEM supplemented with 10% FBS. Knockdown efficiency was validated by quantitative real‐time PCR (qRT‐PCR) at 24 h post‐transfection and by Western blot analysis at 48 h.

### 4.9 Plasmid‐Mediated Overexpression

To investigate the functional effects of SOX4 and TFEB, we performed transient overexpression in KGN cells using expression plasmids. Full‐length human SOX4 or TFEB cDNA was cloned into the pcDNA3.1(+) vector. Cells were seeded in 6‐well plates at a density of 2 × 10^5^ cells per well and transfected the following day using Lipofectamine 2000 (Invitrogen) according to the manufacturer's protocol. Initial attempts to overexpress TFEB with 2–4 µg plasmid DNA per well resulted in pronounced cytotoxicity, consistent with TFEB's role in inducing autophagy. To mitigate this, the TFEB plasmid dose was titrated down to 1 µg per well, which substantially reduced cell death while still enabling detectable transgene expression. For the *SOX4* gene, 2 µg of plasmid per well was used without observable toxicity. Cells were harvested 48 h post‐transfection for qRT‐PCR and Western blot analysis to assess overexpression efficiency.

### 4.10 Rescue Experiments

To assess the functional domains of SOX4 required for maintaining chromatin accessibility under iron overload, we generated KGN cell lines transiently expressing either wild‐type (WT) SOX4, a DNA‐binding–deficient mutant in the high‐mobility group (HMG) domain, or a transactivation domain (TAD) deletion mutant. Cells were treated with ferric ammonium citrate (FAC, 1.5 mm) for 72 h to induce iron overload, followed by ATAC‐seq analysis. Briefly, after FAC treatment, nuclei were isolated and subjected to tagmentation using the Nextera Tn5 Transposase, and libraries were prepared following standard protocols. Sequencing was performed on an Illumina NovaSeq platform, and chromatin accessibility was quantified as reads per peak normalized to total library size. Rescue efficiency was evaluated by comparing ATAC‐seq signal intensity at differentially accessible regions between FAC‐treated cells expressing WT or mutant SOX4 vs. empty vector controls.

### 4.11 Dual‐Luciferase Reporter Assay

A 3363 bp genomic fragment spanning the SOX4 promoter region containing the canonical CLEAR motif (GTCATATGTT) was PCR‐amplified and cloned upstream of the firefly luciferase gene in the pGL3‐Basic vector (Tsingke). A mutant reporter construct was generated by site‐directed mutagenesis, in which the core CLEAR motif was disrupted (mutated to TAGTCTAAAG). KGN cells were seeded in 24‐well plates and co‐transfected with either the wild‐type or mutant SOX4 promoter reporter plasmid, a TFEB expression plasmid (or empty vector control), and a Renilla luciferase plasmid (pRL‐TK) for normalization, using Lipofectamine 2000 according to the manufacturer's instructions. At 36 h post‐transfection, cells were lysed, and firefly and Renilla luciferase activities were measured sequentially using the Dual‐Luciferase Reporter Assay System (Promega). Firefly luciferase activity was normalized to Renilla activity, and results are presented as fold change relative to the empty vector control.

### 4.12 Western Blot Analysis

Cells were lysed with ice‐cold radioimmunoprecipitation assay lysis buffer (CWBio, China). Protein concentration was then measured using a BCA protein assay kit (Beyotime, China). Equal amounts of protein (20 µg per lane) were separated by 10% SDS‐PAGE (Beyotime) according to the manufacturer's instructions, then transferred onto PVDF or nitrocellulose membranes. Membranes were blocked with 5% bovine serum albumin (2 g; BD, USA) and 40 mL of Tris‐buffered saline buffer with Tween‐20, followed by incubation with primary antibody at 4°C overnight. GAPDH was used as a loading control in all experiments. Protein band intensities were quantified using ImageJ software (NIH, USA).

### 4.13 Co‐IP and Co‐IP Mass Spectrometry

Co‐immunoprecipitation studies were performed using a Nuclear Complex Co‐IP Kit (Active Motif, 54001) and Protein G Dynabeads (Thermo Fisher Scientific, 10009D). Each immunoprecipitation was performed using 100ug of nuclear extract. Input material was loaded as a control, with 1× corresponding to 20ug of protein. For mass spectrometry (MS) analysis, immunoprecipitated proteins were resolved by SDS‐PAGE, stained with Coomassie Blue, and the excised bands were subjected to in‐gel trypsin digestion. Peptides were analyzed on a Thermo Fisher Nano1200‐Fusion Orbitrap mass spectrometer. Raw MS data were processed using Spectronaut (Biognosys) for protein identification and quantification. STRING webtool (https://cn.string‐db.org/) was used to construct the protein‐protein interaction (PPI) network.

### 4.14 DNA Pull‐Down

DNA pull‐down assay was performed with a DNA pull‐down kit (Gzscbio, Guangzhou, China). Briefly, a genomic fragment encompassing the SOX4 promoter region was amplified by PCR and labeled with biotin at the 5’ or 3’ end. The biotinylated SOX4 promoter DNA was immobilized onto streptavidin‐conjugated magnetic beads. As controls, parallel reactions were set up using: (i) non‐biotinylated promoter DNA (negative control) and (ii) total protein lysate without DNA incubation (input control, representing the positive reference for detectable proteins). The biotinylated DNA was incubated with protein lysates to pull down potential SOX4 promoter‐binding proteins. After extensive washing to remove non‐specific binders, the bead‐bound protein complexes were eluted and analyzed by silver staining followed by liquid chromatography–tandem mass spectrometry (LC‐MS/MS) for protein identification.

### 4.15 ATAC‐seq

ATAC‐seq was performed as previously described with a few modifications [35]. Briefly, 50 000 fresh cells were resuspended in 50 µL of ATAC‐seq resuspension buffer (RSB; 10 mm Tris‐HCl pH 7.4, 10 mm NaCl, and 3 mm MgCl_2_) containing 0.1% NP40, 0.1% Tween‐20, and 0.01% digitonin and incubated on ice for 3 min. After lysis, 1 mL of ATAC‐seq RSB containing 0.1% Tween‐20 (without NP40 or digitonin) was used to wash the nuclei. The nuclei were resuspended in 50 µL of transposition mix (10 µL 5XTTBL (Vazyme TD501), 5 µL TTE Mix V50, and 35 µL water) and mixed by pipetting up and down 20 times. The transposition reactions were incubated at 37°C for 30 min in a thermomixer. After tagmentation, the sample was purified using 1.8× Ampure XP beads. The ATAC‐seq library was PCR‐amplified for 11 cycles with TAE mix (Vazyme TD501) according to the manufacturer's instructions. The amplified DNA was purified with size selection, quantified, and sequenced on an Illumina sequencing platform.

### 4.16 In Situ Hi‐C

In situ Hi‐C was performed as previously described [36]. Briefly, samples were fixed with a final concentration of 1% formaldehyde and quenched with 0.125 m glycine. Cells were lysed on ice‐cold for 15 min in Hi‐C lysis buffer (10 mm Tris‐HCl pH 8.0, 10 mm NaCl, 0.2% Igepal CA630, 1x protease inhibitor cocktail). Pelleted nuclei were washed once with 1x NEBuffer 2 and incubated in 0.5% sodium dodecyl sulfate (SDS) at 62°C for 5 min. After incubating, water and Triton X‐100 were added to quench the SDS. The chromatin was digested with the MboI restriction enzyme (NEB, R0147). Biotin‐14‐dATP was used to mark the DNA ends, followed by proximity ligation in intact nuclei. After reversal of crosslinks, samples were sheared to a length of ∼300 bp, then treated with the End Repair/dA‐Tailing Module (NEB, E7442L) and Ligation Module (NEB, E7445L), according to the manufacturer's instructions. Biotin‐labeled fragments were pulled down using Dynabeads MyOne Streptavidin T1 beads (Life technologies, 65602). The Hi‐C library was PCR‐amplified for approximately 10 cycles using the Q5 master mix (NEB, M0492L) following the operation manual. DNA was then purified with size selection, quantified, and sequenced on an Illumina sequencing platform.

### 4.17 ChIP‐seq Library PREPARATION

ChIP‐seq was conducted according to Zhu et al. (2019) with minor modifications [37]. Cells were cross‐linked in a final concentration of 1% formaldehyde, followed by quenching with glycine. For BRG1 ChIP‐seq, the cells were dual crosslinked with 1.5 mm EGS (ethylene glycol bis‐succinimidyl succinate) for 20 min and 1% formaldehyde for 10 min. Then cells were lysed with lysis buffer (0.2% SDS; 10 mm Tris ‐HCl, pH 8.0; 10 mm EDTA, pH 8.0; proteinase inhibitor cocktail) and were sheared to fragments of about 300–500 bp using a Bioruptor sonicator (Diagenode). Dynabeads Protein A was washed twice with ChIP Buffer (10 mm Tris‐HCl pH7.5, 140 mm NaCl, 1 mm EDTA, 0.5 mm EGTA, 1% Triton X‐100, 0.1% SDS, 0.1% Na‐deoxycholate, Cocktail proteinase inhibitor) and was incubated with antibody at 4°C for 2–3 h. The fragmented chromatin was transferred to bead‐antibody complex tubes and rotated at 4°C overnight. The beads were washed once with low salt buffer (10 mm Tris‐HCl pH7.5, 250 mm NaCl, 1 mm EDTA, 0.5 mm EGTA, 1% Triton X‐100, 0.1% SDS, 0.1% Na‐deoxycholate, Cocktail proteinase inhibitor) and twice with high salt buffer (10 mm Tris‐HCl pH7.5, 500 mm NaCl, 1 mm EDTA, 0.5 mm EGTA, 1% Triton X‐100, 0.1% SDS, 0.1% Na‐deoxycholate, Cocktail proteinase inhibitor). After crosslinking reversal, a library was constructed following Illumina's instructions as in situ Hi‐C. Antibodies used for ChIP‐seq, including H3K27ac (Abcam: ab4729), Lamin B1 (Abcam: ab16048), BRG1 (Abcam: ab110641), CTCF (Abclonal: A1133), and TFEB (Cell signaling: 37785).

### 4.18 RNA‐seq Data Analysis

Raw sequencing reads were first demultiplexed based on index and adapter sequences, followed by quality filtering using the fastp tool to remove low‐quality reads and adapters. The resulting high‐quality reads obtained were then aligned to the human genome reference sequence (GRCh37 build) using the HISAT2 program (version 2.2.1) with default parameters. To identify the differentially expressed genes, we initially used the featureCounts tool to enumerate the number of uniquely mapped reads assigned to each gene within the human genome. Then, the DESeq2 program was used to identify genes that exhibited differential expression between groups using a threshold value of adjusted *p* values less than 0.05 and an absolute fold change greater than 2 (log2FC ≥ 1). Enrichment pathway analysis of differentially expressed genes (DEGs) was performed using the Metascape webtools.

### 4.19 Pre‐Processing and Quality Control of Sequencing Reads

For the ChIP‐Seq, ATAC‐seq, and Hi‐C libraries, the quality of all libraries was assessed using FastQC. Reads with a mean quality score less than or equal to 30 were removed. Adapters were removed by Cutadapt [38], and fragments with length less than or equal to 30 bp (in the ChIP‐Seq and Hi‐C libraries) were removed.

### 4.20 ChIP‐Seq Data Analysis

Raw ChIP‐Seq reads were aligned to the reference genome using bowtie2 [39] in very‐sensitive mode. Only fragments with both ends uniquely mapped with mapping quality (MAPQ) > 10 were retained using SAMtools [40]. Duplicates were removed with the MarkDuplicates tool from Picard. Peaks were called using the MACS2 callpeak command [41]. Narrow peaks were identified for H3K27ac, CTCF, TFEB, and BRG1 libraries. Fold enrichment over control signal tracks was built using the command bdgcmp in MACS2. Peaks present in both replicates were kept as high‐confidence peaks for each condition. Heatmaps and average binding profile around the peak summit were generated using deepTools. Common and condition‐specific peaks were identified based on pairwise overlaps of peaks using ‘bedtools intersect’ command. ChIP‐seq peaks were annotated using HOMER. For Lamin B1, LADs were called using the enriched domain detector (EDD) peak‐calling algorithm with a gap penalty of 5 [42].

### 4.21 ATAC‐seq Data Processing

All ATAC‐seq reads were mapped to the human reference genome hg19 using bowtie2 in very sensitive mode with option –X 2000 to exclude the largest fragment lengths from the mapping [43]. The uniquely aligned fragments with mapping qualities larger than 10 from both sides were filtered out by samtools [40], and duplicates were removed by the MarkDuplicates in Picard. Fragment length distribution and reads signal around TSS were analyzed using ATACseeker. Peaks were called using MACS2 with parameters ‘–nomodel –shift ‐100 –extsize 200 ‐q 0.05’. Chromatin accessibility signal tracks were built by the bdgcmp module in MACS2 [41]. Differential peaks were identified using DiffBind [44]. To compared ATAC‐seq signal between conditions, we merged peaks to acquire a non‐redundant peak set using BEDTools. Reads counts overlapping each consensus peak were computed using HTseq, and then FPKM (fragments per kilobase per million mapped reads) values were calculated for normalization. ATAC‐seq peaks exhibiting the top 5% and bottom 5% decrease were selected based on DiffBind fold change, and these peaks were annotated using HOMER to determine their nearest genes. For up and down‐regulated genes in RNA‐seq, the changes of ATAC signal around TSS were calculated with ‘macs2 bdgcmp’, and the averaged signal changes of the region were calculated (TSS‐500bp∼TSS+500 bp).

### 4.22 Hi‐C Data Processing

Hi‐C reads were processed using the Juicer pipeline [45]. Contact reads mapping to ChrY and ChrM or with MAPQ = 0 were filtered out. The p(s) is the contact frequency distribution over the log_10_ transformed genome linear distances. Compartments were called by analyzing the first eigenvector of the KR normalized contact maps at 100 kb resolution. The compartments with higher gene density were assigned as type A. Compartment strength was calculated using AB / AA + BB. Saddle plots were calculated as previously described [46]. Hi‐C matrix bins were sorted according to the PC1 values. Sorted frequencies were aggregated into 50 groups and averaged to obtain a compartmentalization saddle plot. TADs were called using arrowhead at 50 kb resolution [47]. Shared TADs between different samples were defined as overlapping areas larger than 80% for both samples. Number of CTCF peaks overlapping with TAD boundaries was compared with the number of CTCF peaks overlapping with equal numbers of randomly selected genomic regions having the same length with TAD boundaries. Loops and interactions were detected with HiCCUPS in Juicer Tools at 10 kb resolution [45]. Enhancer or promoter involved loops were those with at least one anchor overlapped with enhancer regions (distal H3K27ac ChIP‐seq peaks) or promoter regions (TSS ± 3 kb). Shared loops were defined as loops with both anchors not shift more than one bin. Aggregate peak analysis was processed with ‘apa’ in Juicer Tools, which generated aggregate heatmaps and average contact signals. Loop strength was calculated using hicstraw software from .hic files. All signal tracks were visualized with the WashU Epigenome Browser [48].

### 4.23 Activity‐by‐Contact (ABC) Model Analysis

We applied the Activity‐by‐Contact (ABC) model to predict enhancer–gene interactions in control and FAC‐treated KGN cells using our ATAC‐seq, H3K27ac ChIP‐seq, Hi‐C, and RNA‐seq data [49]. Briefly, candidate enhancer regions were defined by calling ATAC‐seq peaks with MACS2 (*q* < 0.1), followed by selection of the top 150 000 strongest peaks based on read counts, extension to 500 bp centered on summits, and removal of blacklist regions using *makeCandidateRegions.py*. Enhancer activity was quantified as the geometric mean of normalized ATAC‐seq and H3K27ac ChIP‐seq read counts within these regions using *run.neighborhoods.py*, with quantile normalization applied against the K562 reference (–qnorm). Hi‐C contact frequencies were derived from our in situ Hi‐C data at 5‐kb resolution; power‐law scaling was used to normalize distance‐dependent decay. Finally, ABC scores were computed genome‐wide using predict.py, considering all candidate enhancers within ±5 Mb of each gene's transcription start site. Only links involving expressed genes (TPM > 1) were retained for downstream analysis.

### 4.24 Statistical Analysis

Statistical analysis was performed in R (version 4.3.2) using the dplyr, ggplot2, maftools, and ggpubr packages. Comparisons between two groups used the unpaired two‐tailed Student's *t*‐test or Wilcoxon rank‐sum test; categorical data were analyzed by the Chi‐square test. All tests were two‐sided with α= 0.05. Exact p values are reported in figures or legends; significance thresholds (e.g., ns, not significant; ^*^ *p* < 0.05; ^**^ *p* < 0.01; ^***^ *p* < 0.001) are indicated by asterisks. Data from qPCR, Western blot, and RNA‐seq were derived from n = 3∼5 independent biological replicates, normalized to internal controls, and presented as mean ± SD.

## Author Contributions

C.W.C. conceived and designed the study, led data curation, and drafted the manuscript. F.F.L. contributed to study design, sequencing experiments, data analysis, and co‐drafted the manuscript. Y.Q.W. performed key experiments (investigation), contributed to validation, and co‐drafted the manuscript. G.Y.Y. performed data curation and formal analyses, and co‐drafted the manuscript. J.Y.L. contributed to experimental validation. X.Y.S. and L.Y.W. contributed to methodology development and validation. J.Z. and X.L. provided additional methodological support and validation. Q.X.Z. and H.C. contributed to study conceptualization and provided guidance on clinical data interpretation. H.Y.L. contributed to clinical sample collection and transcriptome sequencing. B.X.X. assisted with data curation and formal analysis. J.F.Z. and H.L.W. contributed to experimental validation. A.M.M. provided senior conceptual guidance and overall project supervision.

## Conflicts of Interest

The authors declare no conflictss of interest.

## Supporting information




**Supporting File**: advs75568‐sup‐0001‐SuppMat.docx.

## Data Availability

All raw sequencing data generated in this study have been submitted to the Genome Sequence Archive in the National Genomics Data Center [50], China National Center for Bioinformation / Beijing Institute of Genomics, Chinese Academy of Sciences under accession number HRA011472, HRA013483, HRA017112, and HRA016626.
