# Supplementary material for: Dose‐Dependent Reprogramming of Chromatin Accessibility by SOX4 Drives the Transcriptional Response to Iron Overload
Source: Adv Sci (Weinh). 2026 May 7;13(43):e21702. doi: 10.1002/advs.202521702 (PMC13336116; doi:10.1002/advs.202521702)
Supplement: Supplementary file 1 — Supporting File: advs75568‐sup‐0001‐SuppMat.docx. [file ADVS-13-e21702-s001.docx]

**Dose-Dependent Reprogramming of Chromatin Accessibility by SOX4 Drives the Transcriptional Response to Iron Overload**

*Feifei Li*********, Yaoqiu Wu, Guangyu Yang, Jingyi Lai, Xiaoyue Sun, Liyan Wang, Xiaoli Li, Jing Zhang, Qingxue Zhang, Hui Chen, Haiyan Lin, Bingxiang Xu, Junfeng Zhang, Hailong Wang,* ***Anming Meng*,*** *Chunwei Cao********

**Supplementary Figures**


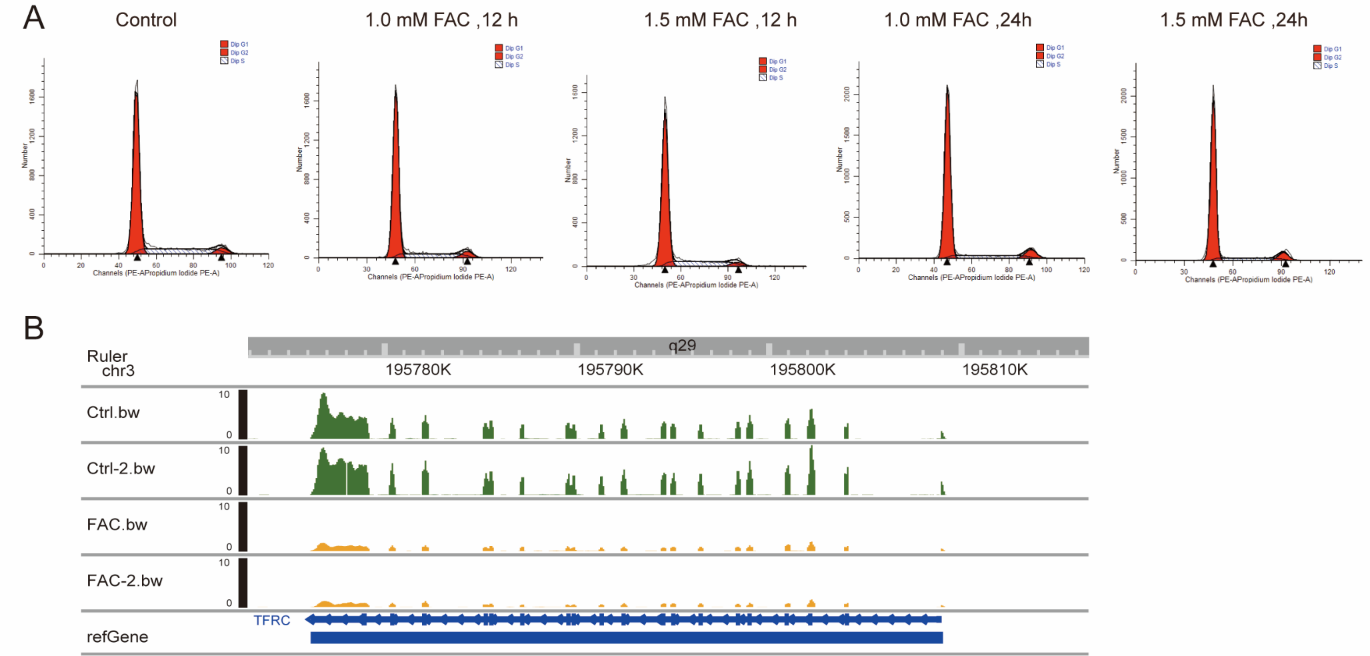


**Figure S1.** Transcriptional impact of iron overload on KGN cells. (A) Flow cytometric analysis displays the distribution of cell cycle. (B) RNA-seq signal of *TFRC* gene.


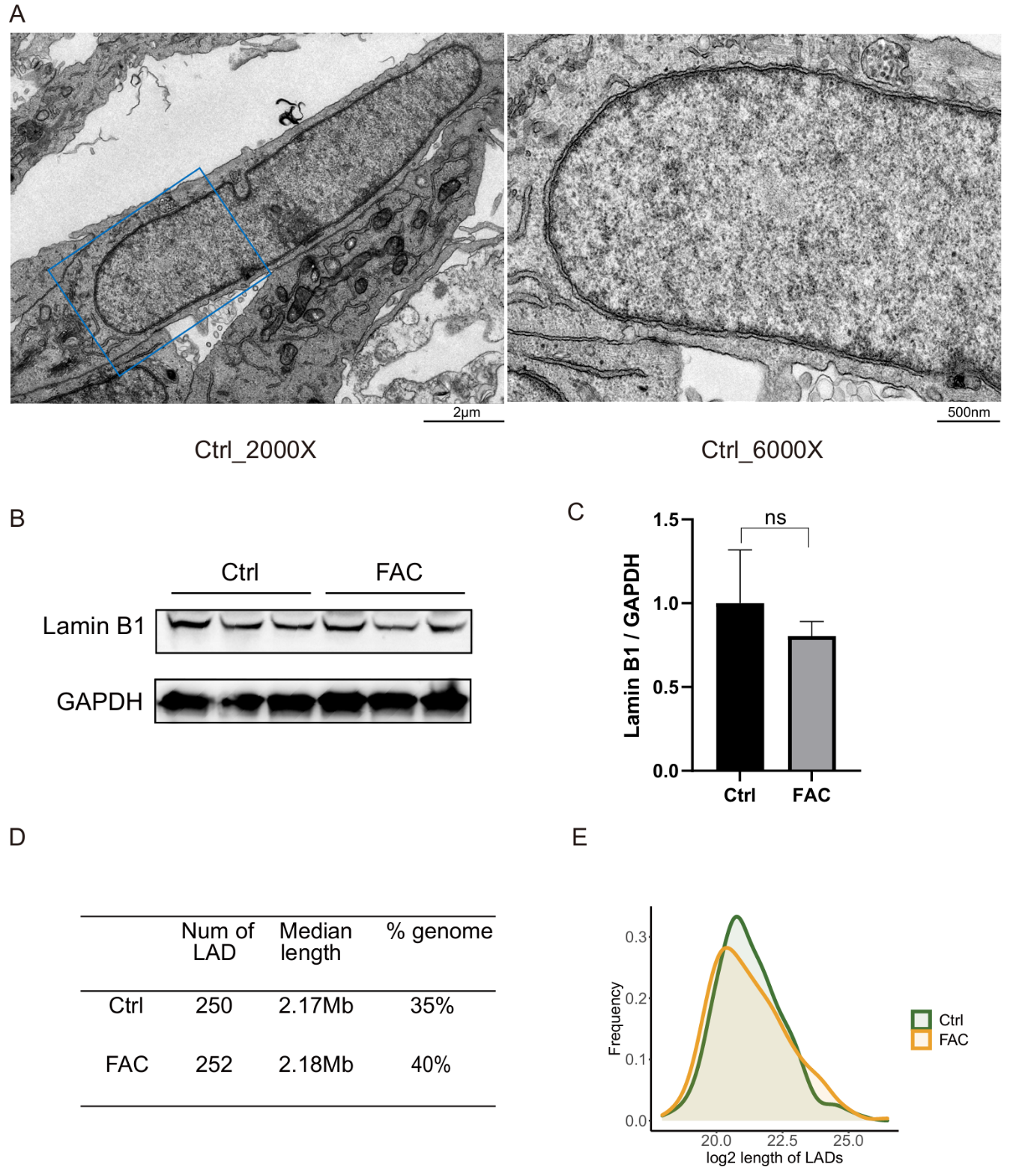


**Figure S2.** Lamin B binding kept stable during iron-overload. (A) Transmission Electron Microscopy (TEM) images of control KGN Cells. The area outlined in blue indicates the region enlarged in the right panel. (B and C) Western blot analysis and quantitation of Lamin B1 protein after FAC treatment (two-tailed unpaired t-test, p = 0.23). Each experiment was repeated three times and the results are presented as means ± SD. Statistical notation used throughout the figure: ns, not significant; * p < 0.05; ** p < 0.01; *** p < 0.001. (D) Statistical analysis of the length and number of LADs detected under different conditions. (E) Length distribution of detected LADs.


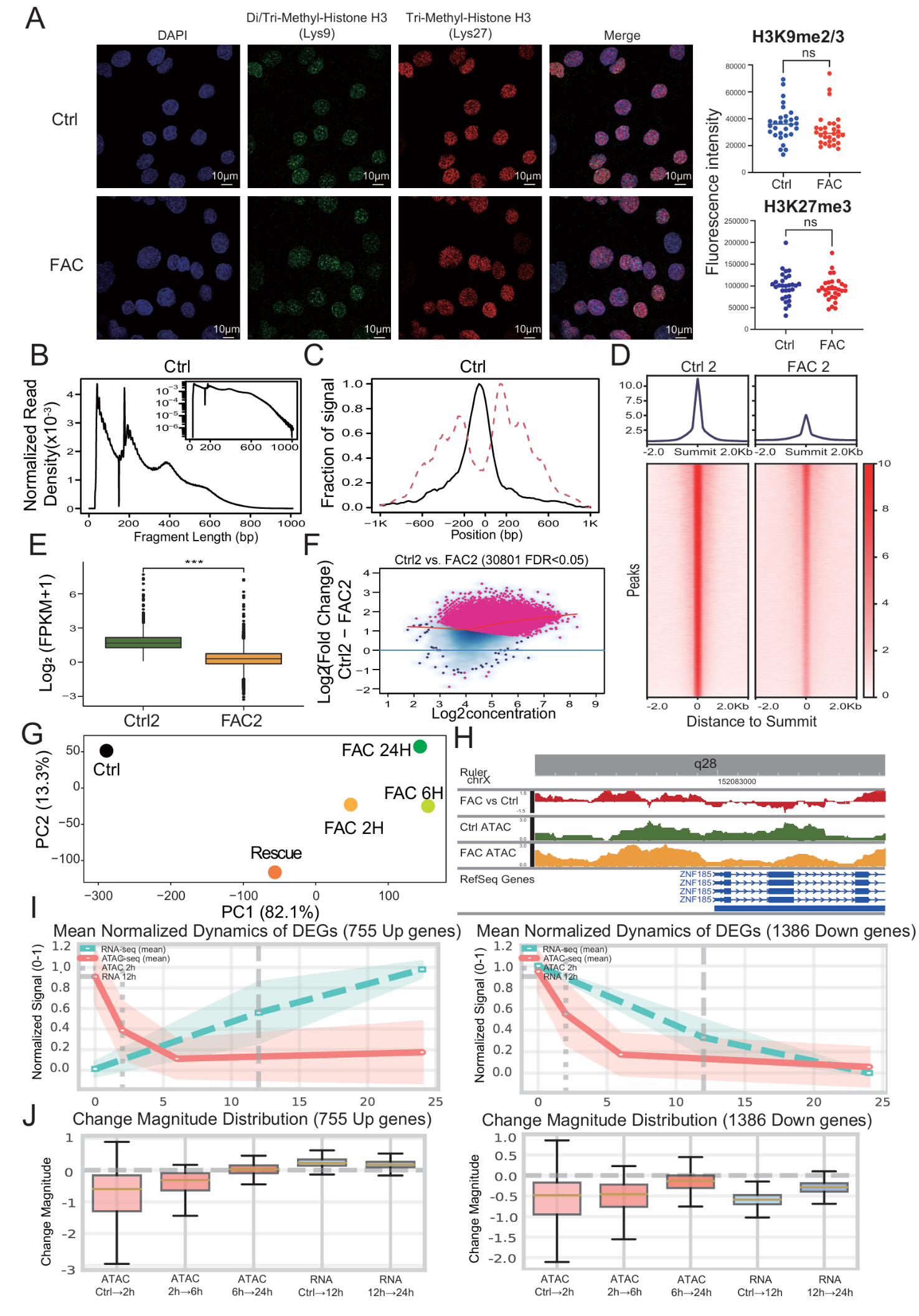


**Figure S3.** Dynamic of chromatin accessibility correlate with transcriptional reprogramming. (A) Immunofluorescence and quantitative comparison of H3K9me3 and H3K27me3 signal before and after iron-overload. Data are presented as mean ± SD (n = 30 cells per group). Statistical significance was determined by unpaired two-tailed Student’s t-test. Statistical notation used throughout the figure: ns, not significant; * p < 0.05; ** p < 0.01; *** p < 0.001. (B) Fragment length distributions of ATAC-seq showed characteristic peaks corresponding to the nucleosome-free regions (< 100 bp) and mono-, di-, and tri-nucleosomes. (C) TSS enrichment plot showing nucleosome-free fragments are enriched at TSS, while mono-nucleosome fragments are enriched at flanking regions. (D-F) Same as figure 3A-C with repeat experiment. In figure E, the data are presented as mean ± SD (n = 42,009 peaks for per condition). Statistical significance was determined by an unpaired two-tailed Student’s t-test. Statistical notation used throughout the figure: ns, not significant; * p < 0.05; ** p < 0.01; *** p < 0.001. (G) PCA analysis of ATAC-seq reads density at peaks of control cells. (H) ATAC-seq profile near promoter of ZNF185 before and after iron-overload. (I) Mean normalized signals (scaled 0-1 per gene) of ATAC-seq (chromatin accessibility) and RNA-seq (steady-state transcript levels) over time for up- and down-regulated genes, respectively. (J) Magnitude of signal changes in consecutive time intervals, illustrating the early plateau of ATAC-seq changes versus the sustained evolution of RNA-seq alterations.


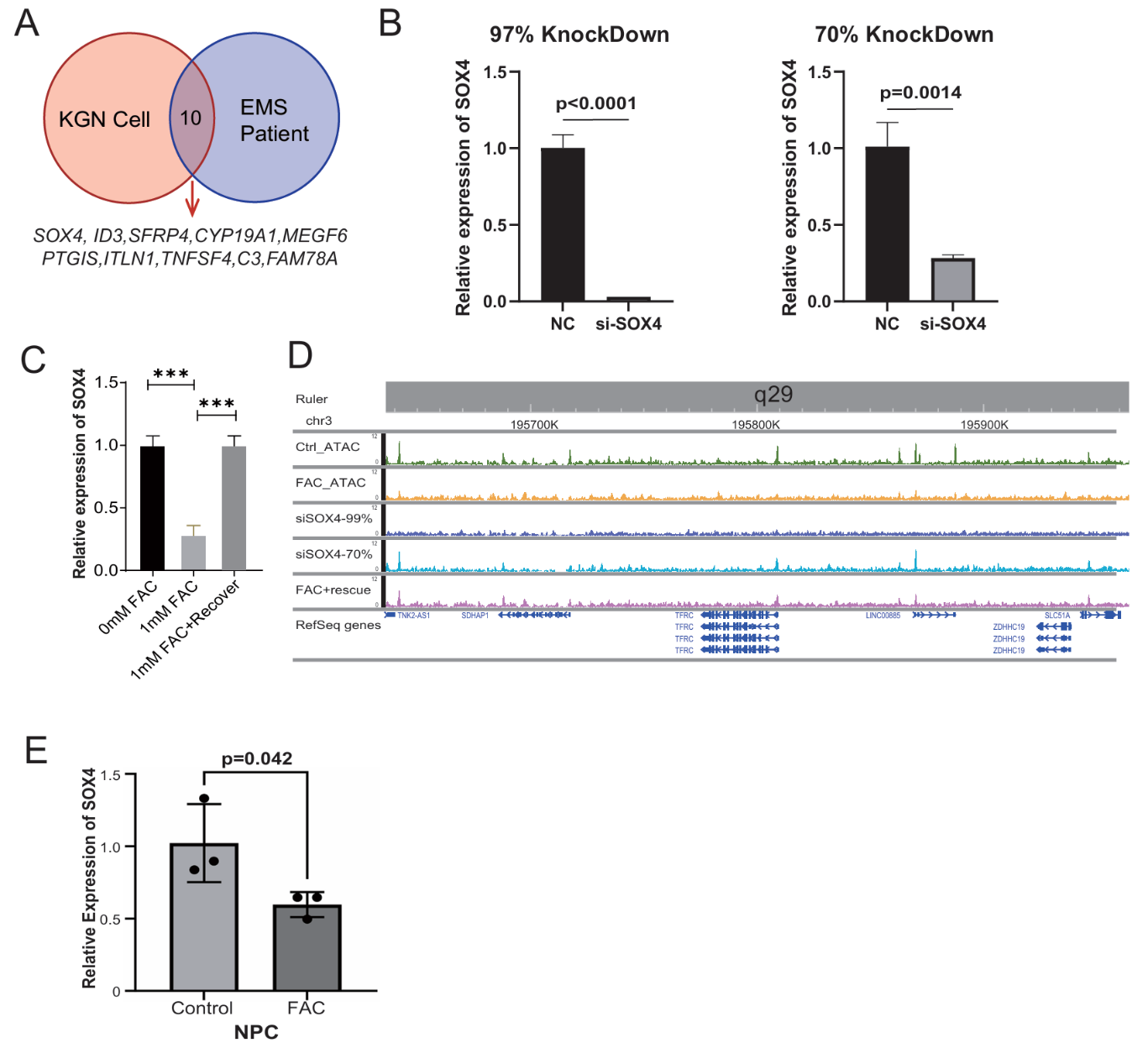


**Figure S4.** SOX4 orchestrates iron-responsive chromatin accessibility. (A) Venn diagram showing the overlap of down-regulated genes in FAC treated KGN cells and in the affected ovary of EMS patient. (B) qPCR validation of the SOX4 knockdown efficiency. (C) SOX4 expression after FAC treatment and recovery (two-tailed unpaired t-test; p < 0.001 for 1 nM FAC vs. 0 nM FAC and for 1 nM FAC vs. 1 nM FAC + recovery). Statistical notation used throughout the figure: ns, not significant; * p < 0.05; ** p < 0.01; *** p < 0.001. (D) ATAC-seq profile surrounding TFRC in conditions of SOX4 knockdown, FAC treatment and rescue after FAC treatment. (E) SOX4 expression after FAC treatment in NPC cells. Each experiment was repeated three times and the results are presented as means ± SD. Statistical comparison for SOX4 was by two-tailed unpaired t-test (p = 0.042).


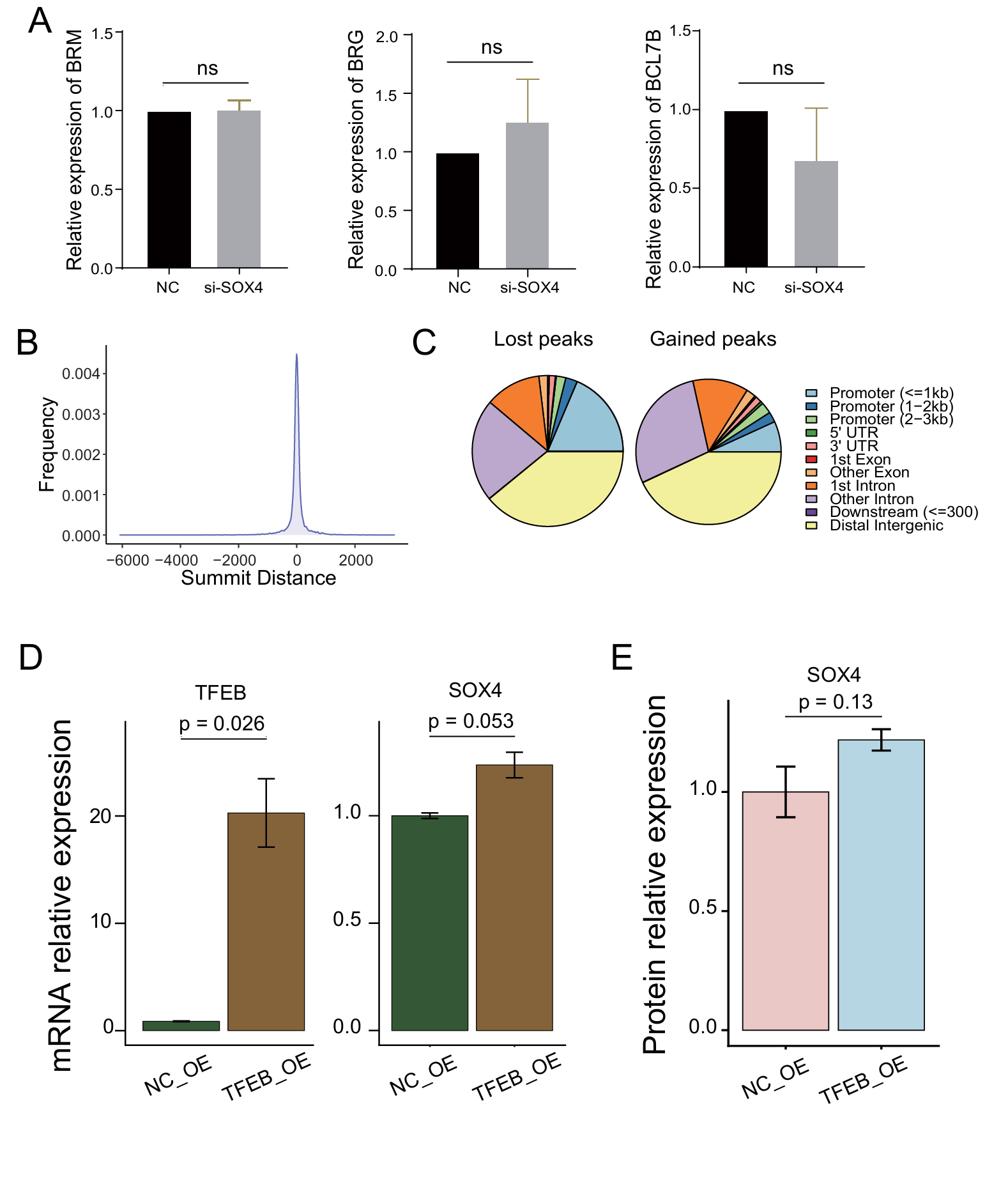


**Figure S5.** Effects of SOX4 knockdown on SWI/SNF binding and validation of TFEB-mediated SOX4 induction. (A) qPCR analysis of BRM, BRG1, and BCL7B mRNA levels upon SOX4 knockdown (two-tailed unpaired t-test; p = 0.79, 0.28, and 0.17, respectively). Each experiment was repeated three times and the results are presented as means ± SD. Statistical notation used throughout the figure: ns, not significant; * p < 0.05; ** p < 0.01; *** p < 0.001. (B) Distribution of distances between BRG1 binding summits and the nearest ATAC-seq summits. (C) Distribution of lost (left) and gained (right) BRG1 peaks over genome elements after SOX4 knockdown. (D) qPCR validation of SOX4 and TFEB mRNA expression following TFEB overexpression. (E) Western blot validation of SOX4 protein expression after TFEB overexpression. qPCR, western blot were repeated three times and the results are presented as means ± SD. Unpaired two-tailed Student’s t-test was used for significant comparison and exact p values are indicated in the figures.


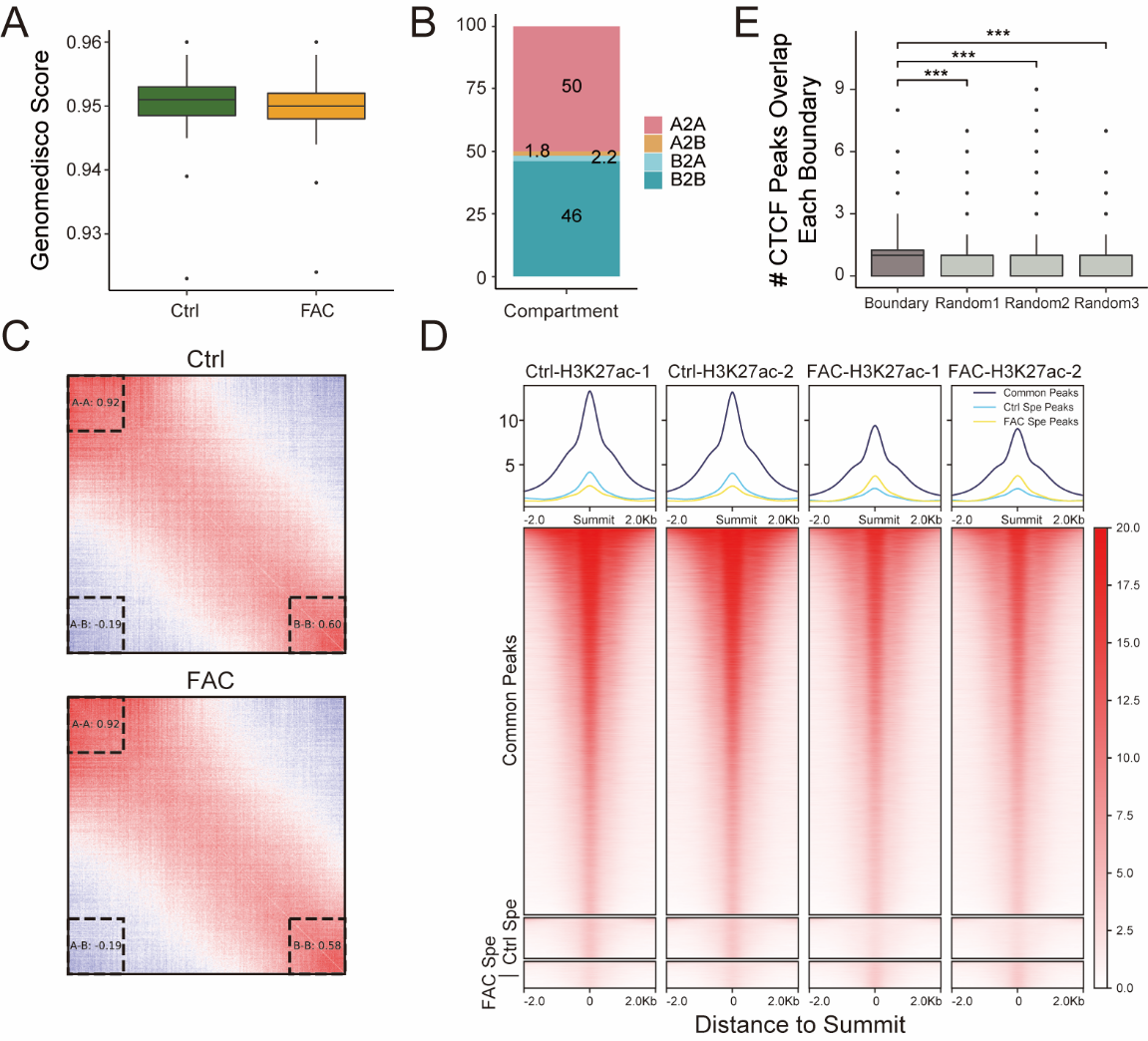


**Figure S6.** Large-scale 3D genome conformation kept stable in iron-overload of KGN cells. (A) GenomeDisco scores showing the reproducibility of the Hi-C libraries. (B) Proportions of genome regions which switched compartments. (C) Compartmentalization saddle plots of different conditions. Numbers represent relative interaction strength between compartments. (D) Heatmap illustrating H3K27ac intensity in common and condition specific peaks for control and FAC-treated KGN cells in each replicate. (E) Boxplot showing number of CTCF binding sites overlapped with TAD boundaries and compared with random selected regions having same length as TAD boundaries. Data are presented as mean ± SD (n = 4,250 boundaries). Statistical significance was determined by unpaired two-tailed Student’s t-test. Statistical notation used throughout the figure: ns, not significant; * p < 0.05; ** p < 0.01; *** p < 0.001.


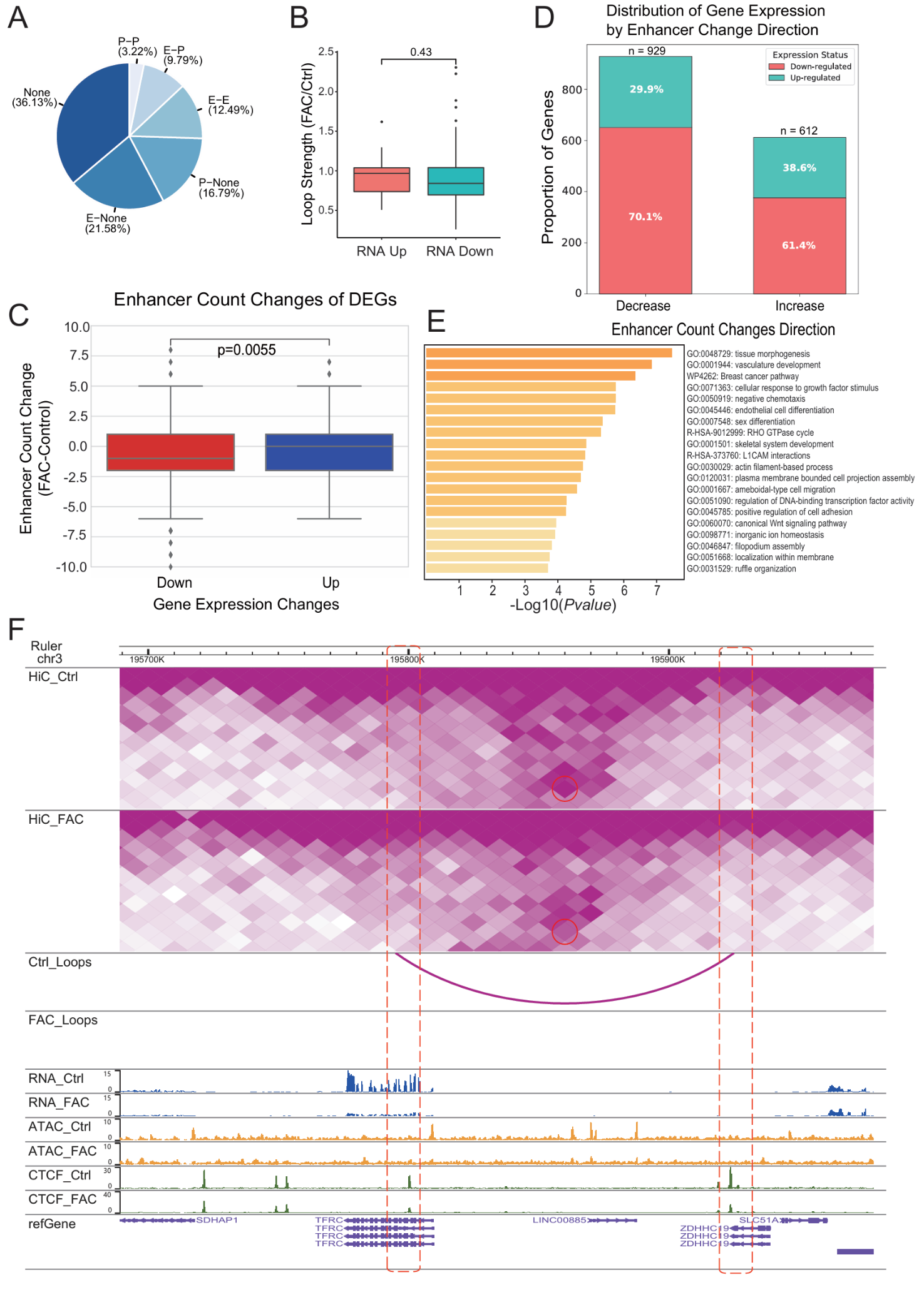


**Figure S7.** Chromatin loops fine-tune the expression of iron-responsive genes. (A) The distribution of identified interactions in control KGN cells. E, enhancer; P, promoter; None, neither enhancer nor promoter. (B) Changes of loop strength for loops connecting promoters of upregulated (n = 678) and downregulated (n = 1,310) genes after FAC treatment. Data are presented as mean ± SD. Statistical significance was determined by unpaired two-tailed Student’s t-test. (C) Boxplot showing the change in ABC model-predicted enhancer count (FAC-treated vs. control) for differentially expressed genes (DEGs), stratified by expression direction. Data are presented as mean ± SD for upregulated genes (n = 678) and downregulated genes (n = 1,310). Statistical significance was determined by an unpaired two-tailed Student’s t-test. (D) Stacked bar chart depicting the proportion of down- and up-regulated genes among those that lost (n = 929) or gained (n = 612) enhancers predicted by ABC model. Statistical significance was assessed using a Chi-square test. (E) Enriched pathways for DEG genes that connected by condition-specific loops. (F) Schematic of Hi-C contact map, identified loops, RNA-seq, ATAC-seq, CTCF ChIP-seq maps near the TFRC gene, which showed control specific loop mediated by CTCF, accompanying decreased CTCF binding in both loop anchors, as well as down-regulation of TFRC after FAC treatment.


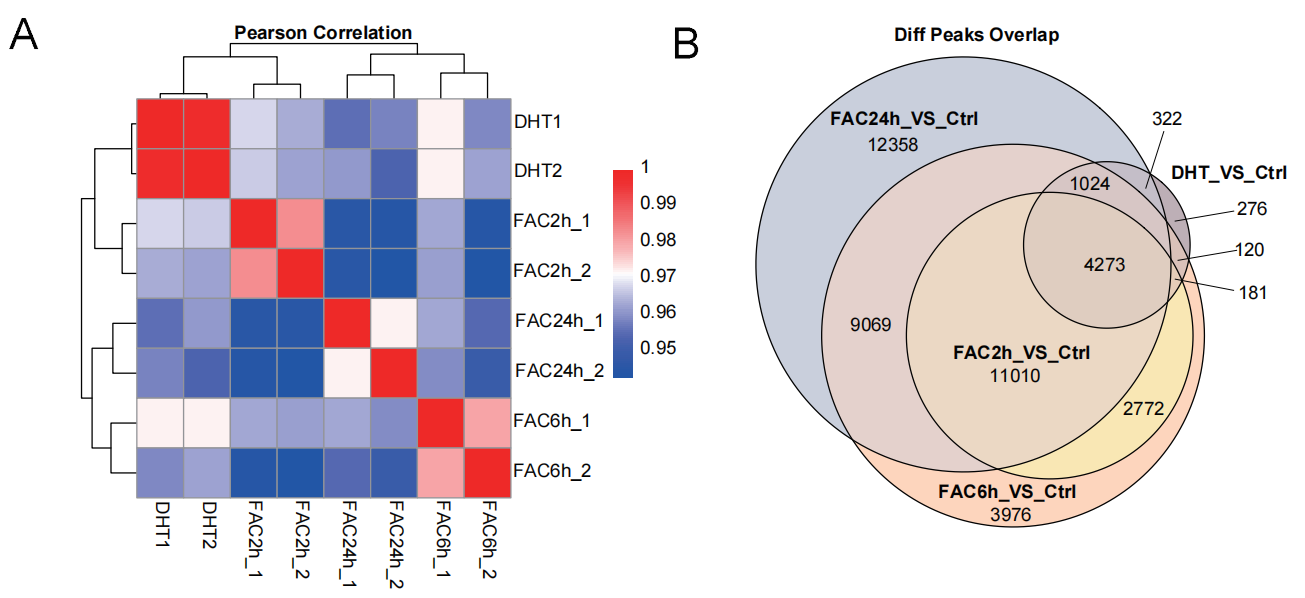


**Figure S8.** Correlation between iron overload and hyperandrogen stimulation models. (A) Pearson correlation heatmap of the counts matrix from all treatment groups after batch effect removal. (B) Venn diagram showing the overlap of downregulated ATAC peaks under different treatments.

**Supplementary Tables**

**Supplementary Table 1.** Summarization of ATAC-seq data in this study

| Samples | Final reads | Number of peaks | Final peaks |
| --- | --- | --- | --- |
| ATAC-KGN-1 | 29,185,196 | 52180 | 42741 |
| ATAC-KGN-2 | 23,150,202 | 58501 |  |
| ATAC-KGN-FAC-1 | 40,017,082 | 10993 | 5626 |
| ATAC-KGN-FAC-2 | 30,469,938 | 9586 |  |
| ATAC-FAC2h-1 | 52,681,176 | 34841 | 24905 |
| ATAC- FAC2h-2 | 40,497,642 | 31774 |  |
| ATAC- FAC6h-1 | 39,679,504 | 14332 | 6113 |
| ATAC- FAC6h-2 | 43,259,310 | 7612 |  |
| FAC-Rescue-ATAC-1 | 20,613,456 | 30923 | 19082 |
| FAC-Rescue-ATAC-2 | 15,262,952 | 25893 |  |
| siSOX4-0.97-ATAC1 | 32,679,380 | 216 | 192 |
| siSOX4-0.97-ATAC2 | 39,211,056 | 586 |  |
| siSOX4-0.7-ATAC-1 | 31,084,250 | 43851 | 27713 |
| siSOX4-0.7-ATAC-2 | 38,410,586 | 34449 |  |
| ATAC-KGN-CTRL-1 | 24,257,606 | 26086 | 17441 |
| ATAC-KGN-CTRL-2 | 36,121,848 | 24488 |  |
| ATAC-KGN-DHT-1 | 70,636,716 | 16214 | 9103 |
| ATAC-KGN-DHT-2 | 29,636,196 | 12597 |  |
| ATAC-KGN-FAC-72h-1 | 6,931,732 | 5326 | 179 |
| ATAC-KGN-FAC-72h-2 | 41,159,016 | 336 |  |
| ATAC-KGN-FAC-SOX4-1 | 32,847,368 | 14873 | 12416 |
| ATAC-KGN-FAC-SOX4-2 | 33,516,996 | 29810 |  |
| ATAC-KGN-FAC-SOX4-DHMG-1 | 52,997,000 | 694 | 228 |
| ATAC-KGN-FAC-SOX4-DHMG-2 | 79,044,376 | 310 |  |
| ATAC-KGN-FAC-SOX4-DTAD-1 | 19,859,976 | 353 | 163 |
| ATAC-KGN-FAC-SOX4-DTAD-2 | 24,115,274 | 341 |  |

**Supplementary Table 2.** Hi-C data summarization

| Samples | Total reads | Total read counts | Alignable | valid pairs | Non-redundant valid pairs | 2 rep valid pairs | Inter-chromosomal | Intra-chromosomal >20kb | Intra-chromosomal <20kb | intra/inter |
| --- | --- | --- | --- | --- | --- | --- | --- | --- | --- | --- |
| KGN-1 | 319,350,986 | 95,805,295,800 | 312,638,051 | 179,261,597 | 153942329 | 308,211,870 | 26,449,606 | 101,608,484 | 25,884,239 | 4.82 |
| KGN-2 | 344,437,566 | 103,331,269,800 | 338,303,932 | 184,515,070 | 154269541 |  | 26,710,129 | 102,643,404 | 24,916,008 | 4.78 |
| FAC-1 | 319,950,092 | 95,985,027,600 | 314,209,064 | 179,894,590 | 147725877 | 291,024,416 | 28,797,719 | 96,264,625 | 22,663,533 | 4.13 |
| FAC-2 | 333,795,832 | 100,138,749,600 | 325,217,850 | 178,874,421 | 143298539 |  | 24,657,874 | 94,031,416 | 24,609,249 | 4.81 |

**Supplementary Table 3.** Down-regulated genes connected by control-specific loops

| Samples | Final reads | Number of peaks | Final peaks |
| --- | --- | --- | --- |
| TFRC | 2.270956119 | 8.24E-35 | 5.00E-30 |
| NEBL | 2.194160017 | 5.88E-10 | 3.16E-07 |
| FPR1 | 1.63343287 | 4.78E-10 | 2.73E-07 |
| PDE5A | 1.613312049 | 1.36E-06 | 0.000142382 |
| RHOBTB1 | 1.496514337 | 4.81E-16 | 2.08E-12 |
| ANK3 | 1.492469474 | 4.30E-08 | 1.06E-05 |
| HEY1 | 1.439331702 | 2.46E-08 | 6.78E-06 |
| RAB17 | 1.416784186 | 1.09E-05 | 0.000695012 |
| CDC14A | 1.305204754 | 1.73E-07 | 2.98E-05 |
| PTGIS | 1.260028484 | 1.57E-08 | 4.78E-06 |
| INTU | 1.256267385 | 2.87E-08 | 7.48E-06 |
| HOXA13 | 1.24020962 | 1.19E-07 | 2.28E-05 |
| SEMA6D | 1.220324567 | 1.41E-05 | 0.000851455 |
| SORBS2 | 1.20136376 | 1.82E-05 | 0.001020322 |
| TTN | 1.166639021 | 7.15E-07 | 8.64E-05 |
| EPAS1 | 1.151878431 | 1.10E-14 | 2.67E-11 |
| ITGA1 | 1.14757836 | 2.14E-07 | 3.47E-05 |
| FZD4 | 1.133570123 | 1.08E-08 | 3.59E-06 |
| SLC26A2 | 1.122689274 | 5.69E-13 | 9.08E-10 |
| DGKI | 1.11289135 | 2.07E-05 | 0.001112844 |
| FNIP2 | 1.111585749 | 6.83E-09 | 2.53E-06 |
| BRIP1 | 1.089055031 | 4.55E-09 | 1.75E-06 |
| OSTM1 | 1.08771588 | 4.64E-10 | 2.68E-07 |
| TPM1 | 1.074602532 | 5.93E-16 | 2.40E-12 |
| SMAD6 | 1.043219167 | 3.93E-11 | 3.14E-08 |
| RDH10 | 1.039721598 | 8.71E-09 | 3.05E-06 |
| ZC2HC1A | 1.027254466 | 2.75E-06 | 0.000243955 |
| AKR1B10 | 1.02576365 | 0.000124614 | 0.004277975 |
| PPM1L | 1.007748134 | 0.000184003 | 0.005771348 |
| PPP1R3B | 0.989121099 | 1.46E-08 | 4.54E-06 |
| PPM1H | 0.988638965 | 1.41E-08 | 4.46E-06 |
| RYR3 | 0.972548593 | 3.88E-05 | 0.001786492 |
| TMEM135 | 0.968741553 | 5.41E-07 | 6.97E-05 |
| MEGF6 | 0.96467625 | 2.59E-05 | 0.001315894 |
| CDK6 | 0.948162847 | 1.09E-07 | 2.15E-05 |
| CHML | 0.94392284 | 2.12E-06 | 0.000199157 |
| TRAF5 | 0.932297731 | 1.68E-07 | 2.94E-05 |
| SFRP4 | 0.924936981 | 0.000677812 | 0.015580438 |
| TRIM2 | 0.923998596 | 1.90E-05 | 0.001050409 |
| SYNPO2 | 0.917813583 | 4.40E-08 | 1.08E-05 |
| SYT14 | 0.916053659 | 8.04E-06 | 0.000555177 |
| TNFSF4 | 0.89814721 | 0.000337572 | 0.009207498 |
| FAM160B1 | 0.896844629 | 5.56E-10 | 3.06E-07 |
| SPTB | 0.890277525 | 6.89E-06 | 0.000493523 |
| ZNF561 | 0.885487627 | 2.90E-06 | 0.000254342 |
| SAMD5 | 0.885286436 | 2.30E-06 | 0.000211595 |
| TYW3 | 0.868296306 | 2.66E-07 | 4.07E-05 |
| GOPC | 0.85886262 | 8.11E-07 | 9.50E-05 |
| HIPK3 | 0.856869635 | 1.43E-08 | 4.50E-06 |
| CSAD | 0.852483311 | 0.000520632 | 0.012765586 |
| IREB2 | 0.850745075 | 5.33E-08 | 1.27E-05 |
| DOCK9 | 0.844523352 | 1.94E-05 | 0.001064251 |
| FGD6 | 0.843023579 | 0.000217576 | 0.006533853 |
| CAPN7 | 0.84129045 | 1.84E-07 | 3.10E-05 |
| SEMA5A | 0.838897895 | 0.000143989 | 0.004786029 |
| SLC19A2 | 0.826943933 | 1.38E-05 | 0.000837514 |
| FAM126B | 0.822755003 | 1.67E-05 | 0.000957924 |
| SLC10A7 | 0.818697374 | 0.000116553 | 0.004061543 |
| RGS5 | 0.818207209 | 0.000334344 | 0.009135866 |
| UBR2 | 0.818061992 | 2.88E-06 | 0.000252461 |
| BAG4 | 0.816544124 | 4.02E-06 | 0.000325781 |
| ADAMTS3 | 0.815657421 | 0.001335524 | 0.026074742 |
| PLEKHA1 | 0.810229209 | 4.85E-07 | 6.39E-05 |
| C8orf37 | 0.810174457 | 0.000623222 | 0.014585373 |
| RHOBTB3 | 0.808833316 | 6.89E-08 | 1.55E-05 |
| ANKRD1 | 0.806291789 | 2.33E-06 | 0.000214038 |
| DPP4 | 0.801478911 | 1.99E-07 | 3.29E-05 |
| BMPR1A | 0.797529725 | 2.21E-06 | 0.000204861 |
| MON2 | 0.796568749 | 6.21E-07 | 7.76E-05 |
| PTEN | 0.79035469 | 7.67E-08 | 1.67E-05 |
| MBNL2 | 0.786914221 | 0.000215568 | 0.00649929 |
| DCP2 | 0.781945602 | 3.74E-06 | 0.000307632 |
| ANKRD46 | 0.779040485 | 8.44E-05 | 0.00319221 |
| LRRC2 | 0.777973062 | 6.82E-05 | 0.002715724 |
| ZNF652 | 0.774349465 | 1.43E-05 | 0.00085737 |
| SOS1 | 0.766743164 | 2.40E-06 | 0.000217858 |
| FLRT2 | 0.765571622 | 4.38E-07 | 5.97E-05 |
| ARHGAP18 | 0.762369602 | 6.36E-05 | 0.002578994 |
| ALDH1L2 | 0.756975791 | 0.000182076 | 0.005740614 |
| ARHGAP42 | 0.754313811 | 0.000422561 | 0.010904629 |
| RPS6KA3 | 0.743811586 | 3.78E-06 | 0.000309999 |
| EPB41L5 | 0.739783033 | 0.000840272 | 0.018249811 |
| NBN | 0.739415723 | 1.48E-05 | 0.00087927 |
| MCOLN2 | 0.736295719 | 0.000288617 | 0.008131413 |
| PKD2 | 0.730466723 | 1.53E-05 | 0.000898306 |
| ZNF627 | 0.725757394 | 0.002133746 | 0.037441465 |
| TMEM87B | 0.724532789 | 1.55E-05 | 0.000904646 |
| GPD2 | 0.72230747 | 5.87E-06 | 0.000433902 |
| MPP5 | 0.718553305 | 2.44E-05 | 0.001252729 |
| OFD1 | 0.717043506 | 4.09E-05 | 0.001862254 |
| ZBTB18 | 0.712481719 | 3.52E-06 | 0.000294858 |
| WWP1 | 0.709938611 | 1.79E-05 | 0.001007723 |
| ACAP2 | 0.697507258 | 2.43E-05 | 0.00125218 |
| LGALS8 | 0.69548328 | 9.64E-06 | 0.000635128 |
| FGD4 | 0.69381289 | 0.001813628 | 0.033047901 |
| PPP1CB | 0.678826416 | 4.91E-05 | 0.002132565 |
| RAPGEF2 | 0.678501684 | 1.19E-05 | 0.000749477 |
| RALGAPA1 | 0.677338199 | 0.000206656 | 0.006283683 |
| SLC2A11 | 0.675667889 | 0.001921603 | 0.034558668 |
| CHD1 | 0.670309076 | 0.000149235 | 0.00493087 |
| ARL15 | 0.663619767 | 0.0016749 | 0.031061184 |
| STC1 | 0.663560427 | 0.000374615 | 0.009949444 |
| OSBPL9 | 0.661353768 | 2.51E-05 | 0.001286495 |
| CCDC93 | 0.658152057 | 3.36E-05 | 0.00160404 |
| ROR1 | 0.65656391 | 3.31E-05 | 0.001589666 |
| C2orf42 | 0.655383583 | 0.000216947 | 0.00652465 |
| LRRC8B | 0.653184902 | 0.002356951 | 0.040594835 |
| WDR35 | 0.645518385 | 0.001950991 | 0.034993803 |
| KHDC4 | 0.644328188 | 1.11E-05 | 0.000703522 |
| CNKSR2 | 0.641729951 | 0.000566934 | 0.013609322 |
| ZNF326 | 0.640958138 | 2.63E-05 | 0.001327424 |
| XPO4 | 0.638468171 | 0.00207986 | 0.036731927 |
| MAN1A2 | 0.63826178 | 0.000361668 | 0.009686168 |
| PDZRN3 | 0.63458821 | 0.002663204 | 0.044780092 |
| PIK3R3 | 0.632154755 | 0.000897723 | 0.019222305 |
| EYA3 | 0.631887303 | 0.00229752 | 0.039797214 |
| LGR4 | 0.626867607 | 0.000117786 | 0.004096915 |
| HSD17B11 | 0.621411211 | 0.000580017 | 0.013824772 |
| MALT1 | 0.613937546 | 8.68E-05 | 0.003254732 |
| KYAT3 | 0.599785031 | 0.001393134 | 0.026973787 |
| NRP2 | 0.598241697 | 9.31E-06 | 0.000618637 |
| AFTPH | 0.596703457 | 0.000243987 | 0.007143091 |
| UPF2 | 0.589375706 | 0.000185343 | 0.005792413 |
| TTBK2 | 0.588039482 | 0.001483259 | 0.028321046 |
| VPS36 | 0.586731919 | 0.000416046 | 0.010780757 |

**Supplementary Table 4.** Up-regulated genes connected by FAC-specific loops

| Gene_name | logFC(con/2410)_RNA | PValue_RNA | FDR_RNA |
| --- | --- | --- | --- |
| CORO2B | -1.687247078 | 1.00E-11 | 1.07E-08 |
| PLAT | -0.980425341 | 1.37E-11 | 1.34E-08 |
| NECTIN1 | -0.938191958 | 2.54E-10 | 1.56E-07 |
| GDNF | -0.931701514 | 6.38E-05 | 0.002584649 |
| KCNB1 | -0.85146202 | 0.000282532 | 0.007990069 |
| ETV4 | -0.848086859 | 1.70E-07 | 2.95E-05 |
| POC1A | -0.814760913 | 1.82E-07 | 3.10E-05 |
| TP53I11 | -0.787704518 | 8.46E-08 | 1.79E-05 |
| CRISPLD2 | -0.761106072 | 3.03E-05 | 0.001487474 |
| GOT1 | -0.74643522 | 1.71E-07 | 2.95E-05 |
| DPYSL2 | -0.691201302 | 1.83E-07 | 3.10E-05 |
| EIF6 | -0.67543567 | 7.85E-07 | 9.27E-05 |
| IGFBP2 | -0.673317154 | 0.000342191 | 0.009304187 |
| PSRC1 | -0.641369627 | 1.01E-05 | 0.000661038 |
| TOM1L2 | -0.636464467 | 5.60E-06 | 0.000420587 |
| ETHE1 | -0.630096056 | 0.001396992 | 0.027039871 |
| RPS29 | -0.626237095 | 0.000191971 | 0.005944445 |
| TRIM58 | -0.624320872 | 2.37E-06 | 0.000216166 |
| RNF123 | -0.623083092 | 7.96E-05 | 0.003054165 |
| BCR | -0.617180451 | 1.11E-05 | 0.000706688 |
| POMGNT1 | -0.610313083 | 1.03E-05 | 0.000669592 |
| RPL37A | -0.600243508 | 1.24E-05 | 0.000771859 |
| DPF3 | -0.589410773 | 0.00028296 | 0.00799844 |
